# The evolution of muscle spindles

**DOI:** 10.1113/EP092264

**Published:** 2024-11-04

**Authors:** Robert W. Banks, Uwe Proske

**Affiliations:** ^1^ Department of Biosciences, and Biophysical Sciences Institute Durham University Durham UK; ^2^ School of Biomedical Sciences Monash University Clayton Victoria Australia

**Keywords:** collateral motor innervation, fusimotor innervation, intrafusal fibres, myotatic reflex, primary ending, secondary ending, spindle capsule, stretch receptors, tetrapods

## Abstract

Muscle spindles are stretch‐sensitive mechanoreceptors found in the skeletal muscles of most four‐limbed vertebrates. They are unique amongst sensory receptors in the ability to regulate their sensitivity by contraction of the intrafusal muscle fibres on which the sensory endings lie. Muscle spindles have revealed a remarkable diversity of functions, including reflex action in posture and locomotion, contributing to bodily self awareness, and influencing wound healing. What were the circumstances which gave rise to the evolution of such complex end‐organs? We argue that spindles first appeared in early amniotes and only later in frogs and toads. This was considered an example of convergent evolution. Spindles in amphibians and reptiles are characterised by their simple structure, pointing to key features essential for spindle function. Spindle sensitivity in amphibians and reptiles is controlled by intrafusal fibre contractions evoked by branches of motor axons supplying extrafusal muscle. Modern phylogenetic evidence has revised our views on the origin of birds, placing them closer to the dinosaurs than had previously been thought. Birds are the only group, other than mammals, which has a dedicated fusimotor innervation of spindles, another example of convergent evolution, given the widely different origins of the two groups. One factor that may have played a role here was that both groups are endotherms, allowing motor control to develop further in an optimal internal environment. This, as well as other changes within the spindle, has led to the astonishing sophistication of motor control observed especially in many modern mammals.

## Abstract (highlights)

- **What is the topic of this review?**
  The evolutionary origins and subsequent specializations of muscle spindles in the major groups of tetrapod vertebrates.
- **What advances does it highlight?**
  Modern phylogenomics allows us to demonstrate that proprioceptors characterizable as muscle spindles have arisen twice in evolution – once in amniotes, which then diversified into diapsids (modern turtles, lizards and snakes, and birds) and synapsids (modern mammals) – and again, separately, in the anurans (frogs and toads). The motor innervation of intrafusal muscle fibres in all modern forms includes collateral branches of skeletomotor axons, but birds and mammals have separately evolved specific fusimotor inputs.

## 1 INTRODUCTION

Why should we want to review the subject of the evolution of the muscle spindle? First, because spindles are interesting! Apart from receptors in the eye and inner ear, spindles are one of the most complex of all peripheral sense organs. Add to that, spindles play a wide range of different roles in the body and are, therefore, an important component of the peripheral sensory apparatus. A great deal is now known about mammalian muscle spindles. They have been shown to contribute to a diverse range of body functions; they are responsible for our sense of position and movement (proprioception), contribute to voluntary motor control and spinal reflexes, and are a part of the neural machinery for posture and locomotion. It has recently been shown that they are not limited to contributing to motor control: they are involved in the realignment of bone fractures, regeneration of spinal axons after injury, and spinal alignment (for a review, see Kröger, 2018). For a receptor with such diverse roles, the question arises, what might be its evolutionary origins?

Microscopic structures, such as muscle spindles, do not, of course, have a fossil record. There exist records of fossilised skin, muscle, bone and even feathers, but not of capsular receptors buried deep within the body of muscles. By necessity, with end‐organs such as muscle spindles, we are restricted in our evolutionary account to descriptions of the structure and function of these receptors in living representatives of the different vertebrate groups. As we shall see, the evidence suggests that spindles probably first evolved in the common amniote ancestor of modern mammals, birds and reptiles and appeared again only later in modern amphibians. It means that spindles evolved separately at least twice and that ancestral spindles, in the form in which they first appeared in amniotes, may have been different from what we see today in amphibians and reptiles. In other words, in our search for the ancestral spindle, we risk being misled if we put too much emphasis on what we see in present‐day non‐mammalian vertebrates.

In exploring the origins of spindles, we have worked with the hypothesis that as vertebrates left the water and began to crawl on land, they were confronted with the problems of supporting their body weight and making movements in the face of the force of gravity. First, it was necessary to evolve limbs from fins, and then, the limbs needed to be able to support the weight of the body. This allowed the animal to stand and for its movements to be transformed from a sliding, slithering motion into proper limb locomotion. As part of the new postural demands, it was necessary to evolve limb muscle reflexes. This made it necessary for stretch‐sensitive muscle sense organs like muscle spindles to provide the afferent signals. Therefore, we propose that the need for spindles evolved as a result of the transition from an aquatic to a terrestrial habitat.

The approach we have taken in exploring the evolution of spindles is somewhat different from that in other evolutionary studies. Given that much is known about mammalian spindles, we have used that information to guide us in the search for similar features in non‐mammalian spindles. We have looked backwards and asked, where did that come from? One consequence of taking such a view is the realisation that several important questions about mammalian spindles remain unanswered. It led us to consider that by taking a comparative view, perhaps we could shine a new light on some of these issues.

In recent years, our views about the sequence of evolution of different vertebrate groups have changed, with consequent implications for possible steps in the evolution of muscle spindles. The issue of convergent evolution arises. This is important since it represents the independent emergence, on more than one occasion, of traits that are sufficiently useful to the animal for evolutionary pressures to resort to them repeatedly. This is worth noting because it allows the assignment of importance to a particular trait and that, in turn, throws new light on speculations about its function.

If two different groups hit on the same solution, despite their differences in anatomy and evolutionary background, it suggests that they faced similar environmental challenges. It also suggests that those solutions are useful in a more universal way. That, for example, seems to be the situation, not only with the evolution of the spindle fusimotor supply in birds and mammals, but with the origin of spindles themselves within amphibians and amniotes. In a broader view, it is possible to compare features of the vertebrate spindle receptor with similar proprioceptive mechanisms in arthropods (Tuthill & Asim, 2018). Such an approach reveals some of the underlying principles of sensorimotor control and its adaptation to rather different circumstances.

The key test for the separate origin of a particular trait, present in two disparate groups, is that the common ancestor does not itself possess that trait. The evidence would be that other descendants of the common ancestor do not possess it. The larger the number of such descendants, the less likely it is that the common ancestor possessed the trait, rather than those descendants having lost it.

It is not intended here to provide a detailed review of spindle structure and function. Our intention is to sketch, in a broad outline, the development of only some of the major features. We will restrict ourselves to discussions of the spindle capsule, the intrafusal fibres, as well as the spindle's sensory and motor innervation. Looking at these characteristics across species will hopefully help provide some insight into the role of spindles in the face of changing demands during the evolutionary process.

There is very little information available on the functional roles of spindles in non‐mammalian vertebrates. However, it is known that the muscle stretch reflex, elicited by signals of muscle spindles, exists in both amphibians and reptiles. This is a reflex response of importance for muscle tone, posture and locomotion. It has, therefore, been traced through the different groups and chosen as an example of the important functional role played by spindles.

Why embark on a comparative study? In recent years, studies of comparative anatomy and physiology have almost disappeared. What that means is that in our search for new knowledge, we have tended to focus on ourselves and taken our eyes off other animals, apart from looking for information they might provide that is useful to us. This is a short‐sighted view! It risks alienating ourselves from our environment.

## 2 THE PHYLOGENETIC BACKGROUND

Here, we concentrate on features such as the presence of a capsule, one or more intrafusal fibres, and sensory and motor innervation because their combined presence in a complex peripheral mechanosensory organ has been implicitly accepted as the defining characteristic of a muscle spindle, beginning with the work of Cajal (1995), Sherrington (1894) and Ruffini (1898). Sensory end organs satisfying these criteria have been described for amphibians, reptiles, birds and mammals. In previous reviews of this subject from a comparative morphological point of view, it was assumed that spindles evolved only once in vertebrates (Regaud & Favre, 1904; Barker, 1974). Are the modern forms of spindles interrelated in representing endpoints of a single continuous evolutionary sequence? Indeed, was there even a single origin for ‘the muscle spindle’? In attempting to answer this question, our starting point is the recent reconstruction of evolutionary relationships for jawed vertebrates based on transcriptomic analysis of 100 species, including representatives of all major gnathostome groups (Irisarri et al., 2017). Apart from a single report of a structure described as a muscle spindle in a jaw‐closing muscle (adductor mandibulae) of a teleost fish (Maeda et al., 1983), ‘muscle spindles’ so‐called and with the above characteristics have only been found in tetrapods. The discussion will, therefore, focus on the Tetrapoda.

**FIGURE 1:**
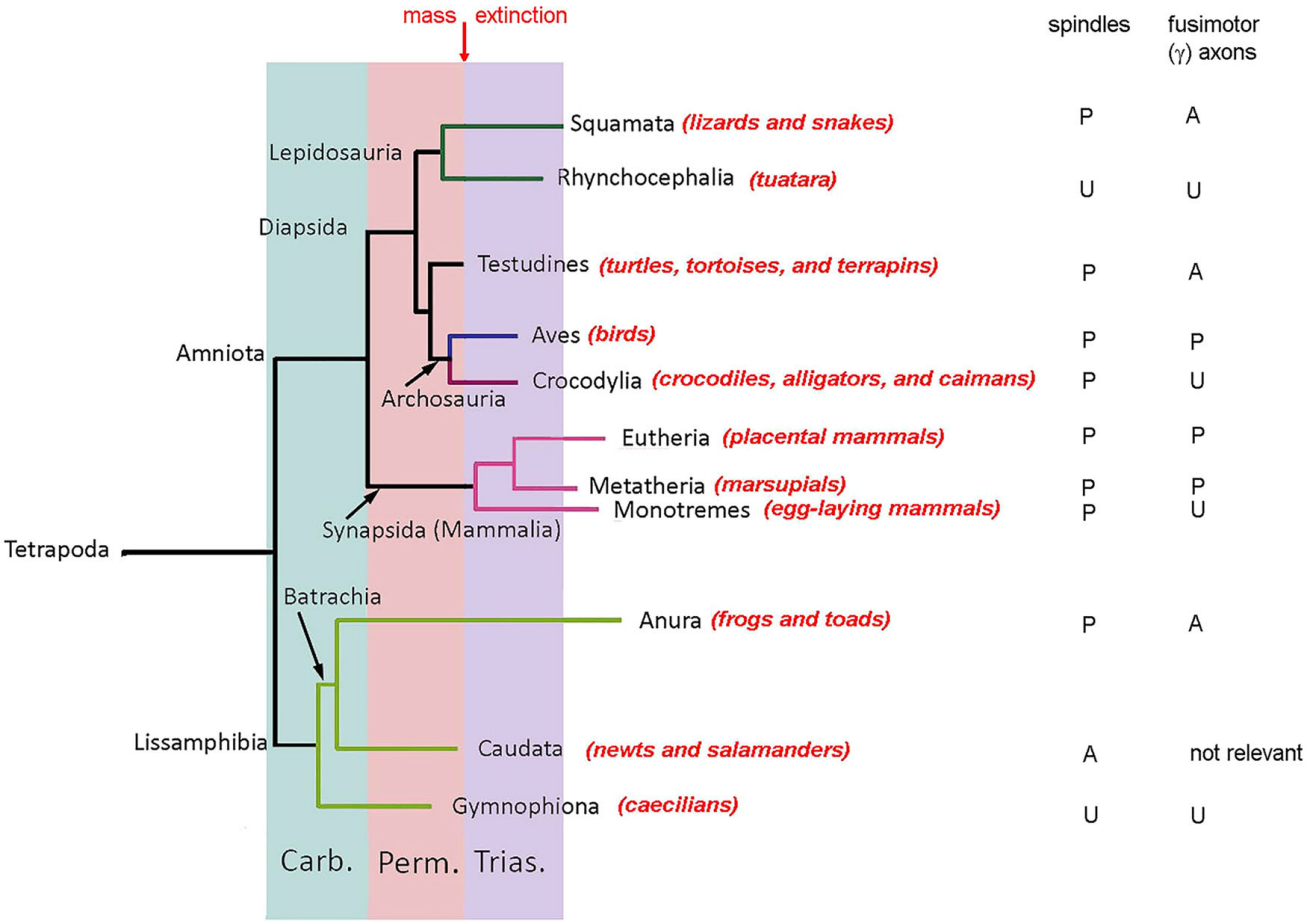
The phylogenetic tree of living forms of tetrapods, simplified and adapted from Irisarri et al. (2017). Vertical coloured bars show the final two periods, Carboniferous (Carb.) and Permian (Perm.), of the Palaeozoic era, and the first period of the Mesozoic era, the Triassic (Trias.), following the end‐Permian mass extinction. The presence (P), or absence (A) of spindles and γ‐fusimotor axons in each modern group are shown in the columns on the right. Lack of information is shown as unknown (U).

The basis of modern phylogenetic systematics consists of identifying a single ancestral form and all of its descendants, all possessing some shared, derived feature(s) (Hennig, 1966). The Tetrapoda is such a group; Figure 1 shows the phylogenetic tree for the tetrapods, simplified from Irisarri et al. (2017). The Diapsida is a group that includes living reptiles and birds, and they have all been found to have muscle spindles. In passing, we note that modern birds are now classified with the theropod dinosaurs (Pickrell, 2014). Similarly, modern mammals are all grouped within the monophyletic Synapsida (Mammalia), and spindles have been found in all three modern synapsid groups (monotremes, marsupials, and placental mammals). It is, therefore, highly likely that the presence of muscle spindles is a common feature of all Amniota, one of the two modern subdivisions of the tetrapods. Within the other monophyletic group, the Lissamphibia, only the Anura (frogs and toads) are known to possess spindles. Despite specific searches for spindles in the Caudata (newts and salamanders), they have not been found (see below), so either the common ancestor (Batrachia) of the Caudata and Anura once possessed spindles and all its caudate descendants have lost them, or, more likely, they never possessed them. In other words, possession of spindles is not a feature of all Batrachia nor, indeed, of the Lissamphibia, though nothing is known about the Gymnophiona (caecilians) in this regard. That, in turn, presents us with our first significant conclusion: that muscle spindles of frogs and toads are likely to have arisen independently and, quite possibly, very much later than in the amniotes.

On the question of the origin of spindles in quadrupeds, there is little evidence in the fossil record. Quadrupeds made their first appearance in the upper Devonian. These were animals living in shallow water and they probably had only a limited ability to walk over land (Clack, 1997). The processes they underwent to become fully land‐based were probably not too dissimilar to those seen in the Anura. There is, therefore, some merit in studying the evolutionary changes undergone by spindles in living amphibians and reptiles and in assuming that a similar sequence of events took place in the Amniota, even though the process undergone by the Anura was likely to have occurred very much later than in the Amniota.

## 3 THE TETRAPODA

This account begins with the portion of the evolutionary tree headed by the Tetrapoda (Figure 1). These are animals, many of whose descendants left the water to take up a life on land. They have four limbs as two bilaterally symmetrical pairs. Phylogenetically, they correspond to the four paired fins (pectoral and pelvic) of their fish ancestors. The Tetrapoda branch into two groups, the Amniota and Lissamphibia. Amniotes are characterised by having an egg equipped with an amnion, an adaptation to enable eggs to be laid on land or for the fertilized egg to be retained within the mother. This distinguishes them from the Lissamphibia, which do not have an amnion.

## 4 LISSAMPHIBIA

The Lissamphibia comprise three groups, the Anura (frogs and toads), the Caudata, (newts and salamanders) and the Gymnophiona (caecilians, snake‐shaped amphibians). It is a clade which has not completely committed itself to living fully on land, and there is an aquatic larval stage that undergoes metamorphosis, to become a land‐dwelling adult. Frogs and toads are further grouped with newts and salamanders as Batrachians as they are more closely related to one another than they are to the Gymnophiona (Figure 1).

In a detailed account of the structure and response properties of sensory receptors in newt and axolotl muscle, Bone et al. (1976) pointed out that no one has ever reported the presence of encapsulated muscle spindles in any urodele muscles, despite repeated searches by different investigators. They described sensory endings lying superficially on muscle fibres, in both newts and axolotls. The endings are stretch receptors, but without any capsule, nor with any structural specialisation in the region of the muscle fibre underlying the sensory ending. Muscles of urodeles contain two kinds of muscle fibres, twitch and tonic, and both are able to receive these sensory endings.

The fact that the endings are always located on the surface of muscles has prompted the suggestion that in a superficial location, a capsule is not necessary. This is because here the endings do not have to be protected from lateral compressive forces acting on them during muscle contraction (Bone et al., 1976). Given that in Batrachians spindles made their first appearance in the Anura, and that Anura spend more of their time out of the water than most urodeles, it raises the possibility that simple stretch receptors were not sufficient for the demands of locomotion on land, and a signal was required that provided more global information about muscle activity. For that, the receptor had to be buried deep within the volume of the muscle, which, in turn, required the protective presence of a capsule.

Urodeles have retained a form of locomotion on land that resembles their swimming motion in water. The axial muscles are large and provide the primary propulsive force for locomotion. By comparison, the limbs are small and weak and tend to be carried forward passively by the undulating motions of the body. It comes as no surprise, therefore, that spindles are absent in urodeles. There is no requirement for generating reflex force in limb muscles to lift the weight of the body off the ground.

Anuran muscles not only have muscle spindles, but stretch receptors like those in urodeles as well (Birchall & Proske, 1977; Ito, 1968). The afferent axons serving these stretch receptors are myelinated and conduct impulses only slightly more slowly than do afferents from spindles (Birchall & Proske, 1977). One possibility is that in anurans, these receptors are a vestige of the more common pattern seen in urodeles and that the urodele receptors represent the precursors of spindles.

## 5 ANURA

Frogs and toads are much more mobile than urodeles; they can crawl, jump, climb trees and burrow in the earth. They are able to generate a series of myotatic (stretch) reflexes in limb muscles (Sassa, 1921) as the animals leave the water and climb onto land. A frog in the water, with its limbs out of contact with obstacles, will always swim. When its feet find contact with solid support, it exhibits diagonal ambulation, flexion of the forelimb and extension of the contralateral hindlimb, allowing it to crawl onto the land (Gray & Lissmann, 1946). The reflex basis of these movements is provided by the stretch reflex, generated by a stretch of passive muscle, giving rise to a reflex contraction. The afferent limb of that reflex is provided by muscle spindles (Matthews, 1972).

Within the Batrachia (Figure 1), the ancestor of the Anura probably separated from that of the Caudata in the Carboniferous period, but their radiation into modern groups occurred up to 100 million years later, most likely in the Jurassic period (Irisarri et al., 2017). Therefore, the amphibian spindle not only has a separate origin from that of the Amniotes but also may have evolved much later. The structure of frog spindles, including ultrastructural details of the sensory and motor terminals, has been reviewed by Barker (1974). Here only the major features of frog spindles will be described, together with an account of some physiological observations. In assessing the various features, it is worth remembering that in this group spindles evolved *de novo*, and that similar structures were probably missing in the immediate ancestors. [Correction added on 24 June 2025 after first online publication: The word “Cretaceous” has been replaced with “Carboniferous” in the first sentence of this paragraph]

**FIGURE 2:**
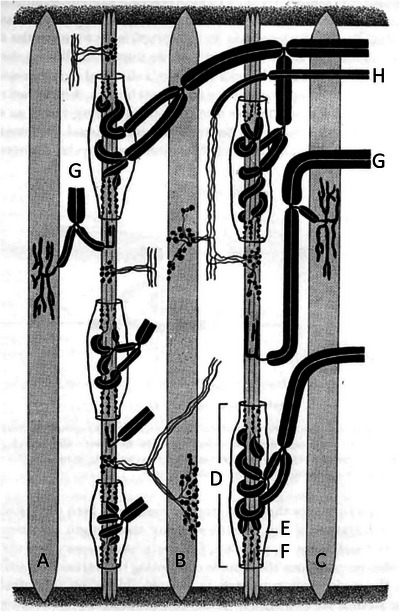
Schematic representation of a spindle in toe muscle of the frog. There are two spindle systems lying between extrafusal fibres, (A), (B) and (C). Fibres (A) and (C) are twitch muscle, (B) is tonic muscle, identifiable by their motor endings, plate for twitch muscle, grape for tonic muscle. The motor endings on the intrafusal fibres of the two spindles are also of two types since they are collaterals of the extrafusal motor axons, (G) twitch axons and (H) tonic axons. The spindle sensory region (D) consists of a capsule (E), which is penetrated by the afferent axon to terminate on the intrafusal fibres (F). Redrawn from Gray (1957).

Muscle spindles in frogs and toads are restricted in their distribution to abdominal, pectoral and limb muscles. They are absent in axial muscles. Frog spindles consist of a bundle of specialised muscle fibres, the intrafusal fibres, enclosed in a connective tissue capsule. The intrafusal bundle typically runs the length of the muscle from the tendon of origin to the tendon of insertion. There are two types of intrafusal fibre, one larger and longer than the other. Both are systematically smaller than the adjacent extrafusal fibres. The spindle capsule is thin‐walled and the intrafusal fibres are loosely enclosed within it, suspended in the periaxial space by struts of connective tissue. A diagrammatic representation of a frog spindle is shown in Figure 2.

In the central capsular region of the spindle, where the sensory terminals are located, the surface of the intrafusal muscle fibres is thrown into a series of irregular folds. Here, there is a network of interstitial spaces filled with collagen, the reticular zone (Katz, 1961), in contrast to the compact zone on either side, where the intrafusal fibres resume their normal appearance. Micrographs of the sensory ending show the presence of chains of bulbs, linked by strands of axoplasm. The nerve bulbs are in contact with muscle fibres in shallow sockets on the muscle surface.

A feature of frog spindles is that the intrafusal bundle may be enclosed by several capsules, each served by an afferent fibre, to form a tandem spindle. Gray (1957) mentioned that sometimes ‘spindle systems’ occur, consisting of bundles of simple and tandem spindles clustered together (Figure 2). Each capsular region receives only a single afferent fibre which branches as it enters the capsule and then terminates on the intrafusal fibres. The spindle motor innervation consists of collaterals of motor axons which also supply extrafusal muscle fibres.

The motor innervation of frog muscle is basically of two kinds, twitch and tonic. Twitch muscle typically responds to a single motor impulse with measurable tension, whereas a tonic motoneuron must be stimulated repeatedly to generate significant tension (Morgan & Proske, 1984). According to Sterling (1974), twitch muscle can be further subdivided into three kinds, slow‐twitch, intermediate twitch and fast‐twitch, and it is the collaterals of motor axons supplying slow‐twitch muscle fibres that innervate the larger intrafusal fibres. The smaller intrafusal fibres are innervated by collaterals of motor axons supplying the tonic muscle. Any one spindle may receive both slow‐twitch and tonic motoneuron collaterals but, as far as we know, any single intrafusal fibre is exclusively innervated by one or other type of motor axons.

Brown (1971a) studied spindles in the iliofibularis muscle of the frog, a muscle known to contain tonic muscle fibres. He perfused the muscle with the depolarising drug suxamethonium, which initiated a sustained contraction of the muscle. About half of the spindles in iliofibularis showed a response to the drug, a weak maintained response, but a large increase in discharge during muscle stretch. Similar treatment of spindles in the sartorius muscle, which does not contain any tonic fibres, gave responses that were weaker than in iliofibularis and the large dynamic response during stretch was missing. It was concluded that some frog spindles received only twitch motor collaterals supplying twitch‐type intrafusal fibres while other spindles received a mixed twitch–tonic innervation. It was the intrafusal tonic fibres that were responsible for the large response to stretch.

**FIGURE 3:**
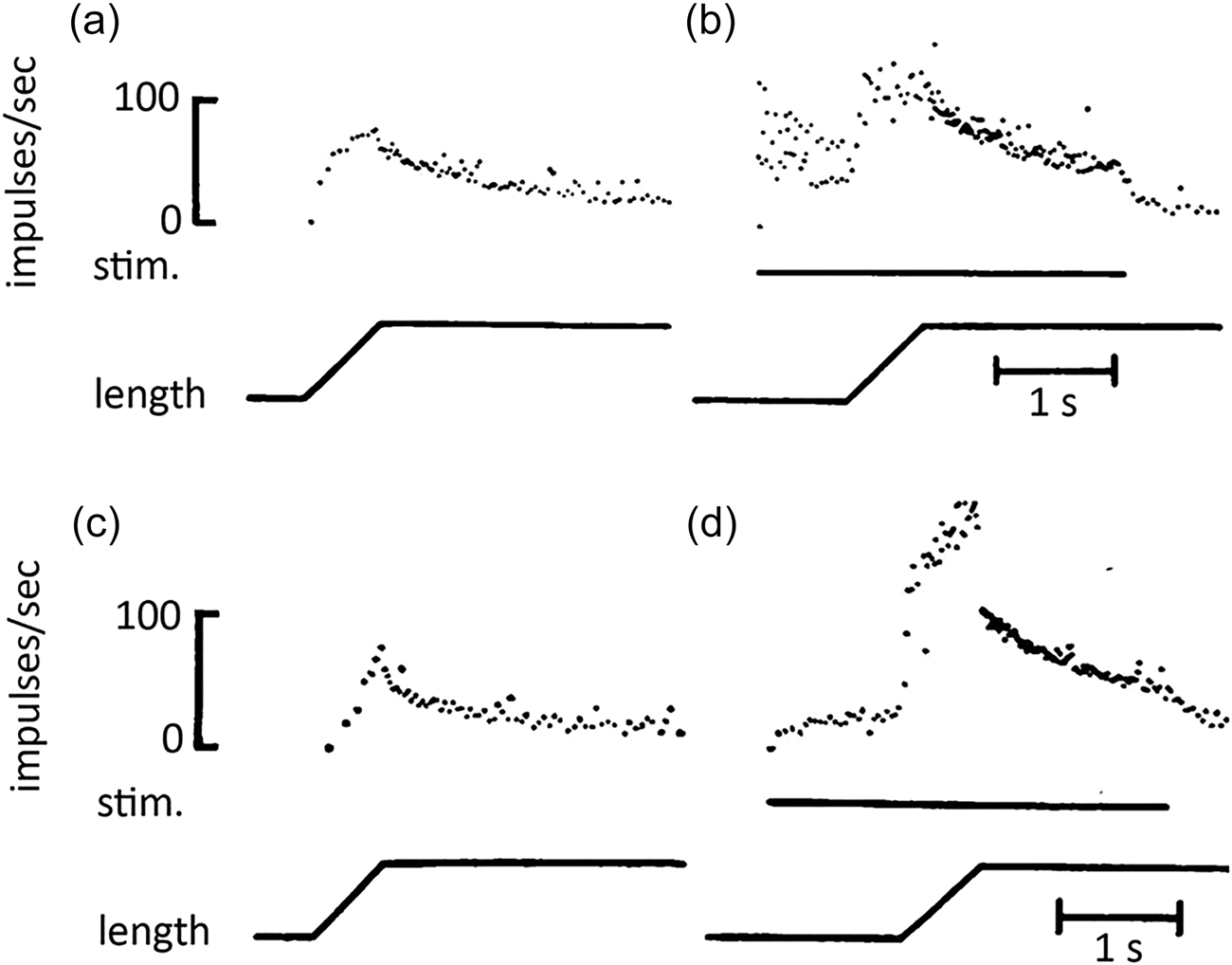
Effects of stimulating twitch and tonic motor axons during stretch of frog spindles. (a) Response of a spindle to a passive stretch (0.5 mm at 2.5 mm s^−1^). Afferent discharge shown as instantaneous frequency display. (b) Combined stretch with stimulation of a twitch motor axon at 25 pulses s^−1^. The bar under the frequency record indicates the period of motor stimulation. (c) Passive response of another spindle. (d) Response during combined stretch and stimulation of a single tonic motor axon at 33 pulses s^−1^. Redrawn from Matthews and Westbury (1965).

This proposal was formally tested by Matthews and Westbury (1965). They showed that stimulating small, tonic motor axons supplying a spindle produced a much larger response to stretch, compared with the response to stretch of the passive spindle. Stimulation of large, twitch motor axons strongly excited the spindle, but the size of the response was little altered by whether or not the muscle was undergoing stretch (Figure 3). The authors suggested that the ability of the spindle to respond strongly to stimulation of small motor axons during stretch meant that this was likely to augment the size of the stretch reflex, given that it was known stretch reflexes were present in frog muscle (Chambers & Simcock, 1960; Sassa, 1921).

To conclude, the spindles making their first appearance within the Lissamphibia in the Anura already have a quite complex structure: the presence of a capsule containing specialised muscle fibres, modified in the region where they receive sensory endings, and the presence of two kinds of motor endings. The two major features seen in mammals, but which are still missing, are a dual sensory innervation and a dedicated spindle motor supply, the fusimotor system.

## 6 THE AMNIOTA

In contrast to the Lissamphibia, the Amniota have three extra‐embryonic membranes, the amnion for embryonic protection (amniotic fluid), the chorion for gas exchange and the allantois for metabolic waste. Amniotes have thicker, keratinized body skin and use costal (rib) respiration. These are all adaptations for living on land. Unlike Lissamphibia, the amniotes do not undergo metamorphosis from an aquatic larval stage. They have an astragalus (talus), an ankle bone which transmits the weight of the body from the lower leg to the foot. Its presence increases the range of motion of the ankle joint and extends stride length in above‐ground locomotion.

All amniotes have muscle spindles. This is another reflection of the group's commitment to living on land. That in amniotes spindles evolved independently of those in anurans seems likely, but it remains to be confirmed.

The Amniota is divide into two major groups, the Diapsida, which includes all of the modern reptiles and birds, and the Synapsida, which consists of mammals. The diapsids comprise the lizards and snakes, the turtles, and the archosaurs, a clade of diapsid reptiles which has birds and crocodiles as its only living representatives. Extinct archosaurs include non‐avian dinosaurs, pterosaurs and extinct relatives of the crocodilians. In the next section, attention will be directed at the Testudines, the turtles and tortoises, whose comparatively simple spindles are a candidate for the ancestral amniote condition.

## 7 THE TESTUDINES

**FIGURE 4:**
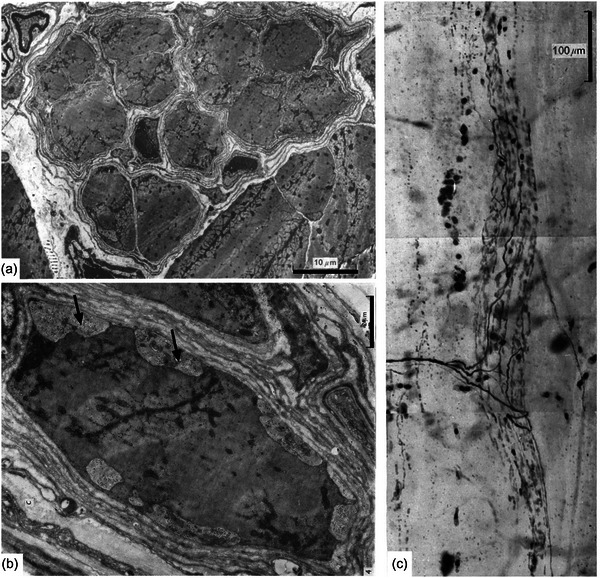
Muscle spindles in extensor digitorum brevis I of the tortoise *Testudo graeca*. (a) Transverse section through the sensory (equatorial) region of a spindle. The multilayered capsule of perineurium encloses 11 intrafusal muscle fibres, all of which are striated throughout the sensory region. (b) Oblique section through the sensory region of an intrafusal muscle fibre bearing several varicose sensory terminals (arrows). The sarcomeric structure is present throughout the sensory region. (c) The sensory region of a teased, whole spindle. Three branches arise from the single, myelinated, sensory axon and distribute varicose sensory terminals to all of the intrafusal fibres by progressively finer branches. (a and b: transmission electron micrographs from Crowe and Ragab (1970b); c: methylene blue preparation from Crowe and Ragab (1970a).

The structure and function of muscle spindles have been studied in several species of terrestrial and freshwater turtles (Crowe & Ragab, 1970a, 1970b; Naeije & Crowe, 1977; Proske & Walker, 1975). The structure of the capsule (Crowe & Ragab, 1970b) is consistent with its being composed of perineurium, as in the spindles of other vertebrates. The capsule extends over only a very short length of the intrafusal fibres (overall about 12% in extensor digitorum brevis I of *Testudo*), there is no periaxial space, and it encloses an average of almost 11 intrafusal fibres in a range of 2–17 (Figure 4a). Most intrafusal fibres extend nearly the full length of the muscle, and therefore are of similar length to the extrafusal fibres. The diameters of intrafusal fibres throughout their lengths are less (mean 10.6 µm) than those of the extrafusal fibres (mean 27.3 µm). Intrafusal fibres attain their maximum diameter at the ends of the capsule, and exhibit a local minimum within the capsule, where they are innervated by the single sensory axon. Only one type of intrafusal fibre has been found either ultrastructurally or by histochemistry (Crowe & Ragab, 1972), though both ‘plate’ and ‘grape’ motor endings were described by Crowe and Ragab (1970a), and were sometimes said to be intermixed in the same spindle. The sensory ending (Figure 4c) consists of many fine axonal branches in contact with the intrafusal fibres that are deeply indented by varicosities along the branches, but that appear otherwise to be relatively unspecialised with sarcomeres throughout the sensory region, no central nuclei and no associated elastic fibres (Figure 4b).

Proske and Walker (1975) recorded the afferent responses of spindles in extensor digitorum longus I of the freshwater tortoise *Chelodina*. At the muscle's in situ length, most spindles had no resting discharge. Their passive length sensitivity was in the range of 2–5 impulses/s/mm and all appeared to be predominantly sensitive to static muscle length rather than rate of length change. Their limited dynamic sensitivity increased essentially linearly at an average rate of about 1.5 impulses/s/mm/s. Mean afferent conduction velocity, measured at room temperature, was 15.3 m/s (range 12.6–18.7 m/s). In their passive properties, all spindle sensory endings of turtles, therefore, seem to resemble long capsule spindles of squamates and secondary endings of mammals.

The intrafusal motor innervation of turtle spindles, whether ending as ‘plates’ or ‘grapes’, is derived from collateral branches of axons to extrafusal fibres (Crowe & Ragab, 1970a). Extrafusal ‘plates’ were thought to characterise twitch fibres, whereas ‘grapes’ were thought to supply slow, or tonic, fibres (Barker, 1974), but despite both types being found in turtle spindles, the intrafusal fibres all appeared to correspond to the extrafusal twitch type on the evidence of their ultrastructure and histochemistry.

**FIGURE 5:**
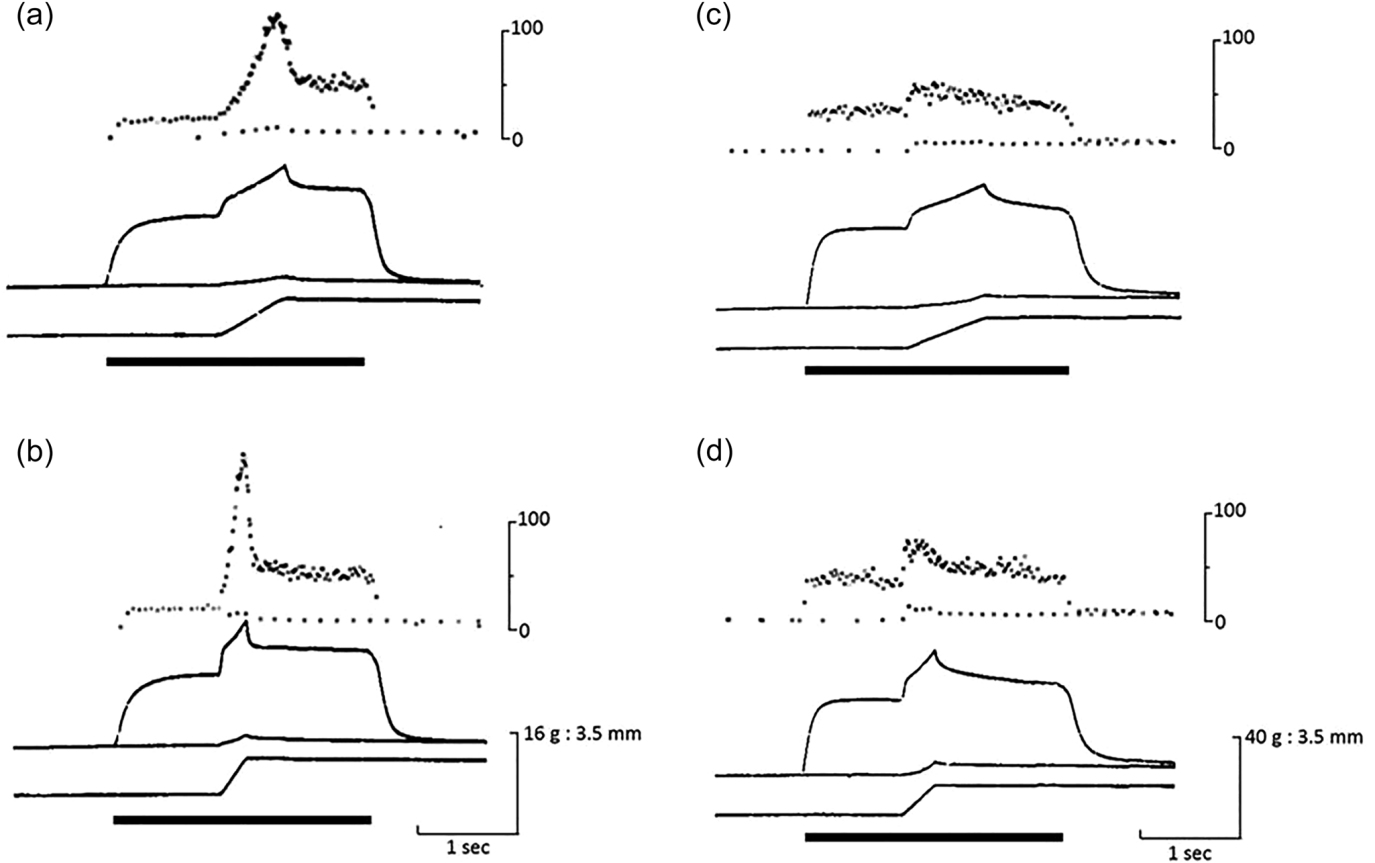
Responses of an extensor digitorum longus muscle spindle in the turtle *Chelodina* to a passive stretch and to the same stretch during stimulation of a motor filament. In (a, b), the motor filament is from tonic muscle, in (c, d) it is from twitch muscle. (a) 1.3 mm stretch at 1.3 mm/s. (b) The same stretch at 5.0 mm/s. (c, d) A stretch of 1 mm at 1.3 (c) and 3.2 mm/s (d). In each panel, the top traces show the instantaneous frequency of spindle discharge to passive stretch superimposed on the response to stretch and motor stimulation. In the trace below is shown passive tension during stretch, superimposed on the active tension during stretch and motor stimulation. The bar at the bottom indicates the period of motor stimulation (40 pulses/s in (a, b) and 50 pulses/s in (c, d)) and the trace immediately above it is the ramp stretch imposed on the muscle. From Proske and Walker (1975).

Proske and Walker (1975) presented evidence for differentiation of the effects of repetitive stimulation of motor filaments on spindle responses to stretch (Figure 5). In the extensor digitorum longus, a mixed muscle containing both twitch and tonic muscle fibres, in response to motor stimulation, for 10 spindles studied all showed only ‘static’ motor effects, while a further 10 spindles showed only ‘dynamic’ motor effects.

One additional spindle showed mixed, static–dynamic effects. It was concluded that the intrafusal fibres of the majority of spindles were of one kind, twitch or tonic, depending on where in the muscle the spindle lay; this determined their responses to muscle stretch and motor stimulation. The effects observed were somewhat reminiscent of the two kinds of motor responses seen in squamates. Naeije and Crowe (1977) reported broadly similar results from the extensor digitorum brevis muscles of three species of turtles, but concluded that ‘the observed range of response patterns did not clearly indicate two distinct categories’.

## 8 THE SQUAMATA

Squamate spindles are characterised by having just one intrafusal fibre, with the rare exception of two fibres, within one capsule. It is likely that the immediate ancestors of the squamates also possessed spindles and that these contained multiple intrafusal fibres within the spindle capsule. Therefore, the evolution of monofibrillar spindles should be seen as a more recent feature of the group. What might be the significance of a spindle with only one intrafusal fibre? It presumably means that the range of spindle responses is limited by the mechanical property of the one intrafusal fibre. Given that in reptiles, as in amphibians, there are two kinds of extrafusal fibres, twitch and tonic, both requiring to be served by afferent feedback from spindles, it means that having a single intrafusal fibre commits the Squamata to have two kinds of spindles, long‐capsule spindles supplied with a twitch intrafusal fibre, and short‐capsule spindles with a tonic intrafusal fibre.

The other feature of squamate spindles, which is also likely to be linked to having only one intrafusal fibre, is the emergence of two different kinds of sensory endings, each with its own characteristic stretch responsiveness. It is interesting to speculate about the evolutionary steps squamate spindles may have undergone to achieve such an outcome. Based on what we know from mammals, it is advantageous for spindles to have sensory endings responsive to both static and dynamic length changes in the muscle. The first step in the evolutionary process aimed at fulfilling this requirement was taken by reptiles, with the evolution of spindles with sensory endings selectively sensitive to static or dynamic length changes. To achieve such a change, resorting to a spindle having only one intrafusal fibre facilitated that process; each spindle was provided with only a single afferent fibre, so it presented the opportunity to have two kinds of spindles.

In frog spindles, the one sensory ending is able to respond to both twitch and tonic intrafusal contractions. Significantly, the static response of frog spindles is enhanced by stimulation of twitch motor collaterals, while the dynamic response is increased by stimulation of tonic motor collaterals (Figure 3). It is possible that the evolutionary process undergone by squamate spindles to create two types of sensory endings may have taken place under the influence of the mechanical properties of the intrafusal muscle fibres on which the sensory endings happened to lie.

Evidence from studies of the early development of mammalian spindles (Milburn, 1984) suggests that the outgrowing sensory and α‐motor axons reach the myotubes, destined to become intrafusal fibres, at similar times. This makes it likely that both are involved in the differentiation of the developing intrafusal fibre. In squamates, the transformation of the sensory region might have only proceeded if the intrafusal fibre on which it lay was already under the influence of one or other of the two kinds of motor axons. The sensory ending that developed would be determined by the contractile characteristics of the developing intrafusal fibre. This would eventually lead to the formation of two kinds of sensory endings.

In squamates, the two sensory‐ending types can be distinguished by their passive responsiveness as well as their responses to motor stimulation. The ending with a pronounced dynamic response to passive stretch has this further enhanced by stimulation of tonic motor axons. The ending with a predominantly static response to passive stretch has its static responsiveness enhanced by twitch motor stimulation. What this suggests is that to have static and dynamic motor responses both processed by the one sensory ending, as seen in frog spindles, maybe a less‐than‐optimal solution; adding sensory terminals that are already biased in their passive responses in the same direction as the motor responses seems to be advantageous for sensorimotor control. The impression is that motor effects, similar to those described for frog spindles, have extended their influence in squamates to lead to development of two distinct sensory ending types whose passive and active responses are complementary. This arrangement reaches a further level of sophistication in mammals, where the spindle primary ending not only has a pronounced dynamic response to passive stretch that can be enhanced by dynamic fusimotor stimulation, but its passive static response can be raised by static fusimotor stimulation.

There have been two comprehensive reviews of squamate spindles (Barker, 1974; Proske and Ridge, 1974). The account here is a précis and update of those accounts.

### 8.1 The sensory ending

**FIGURE 6:**
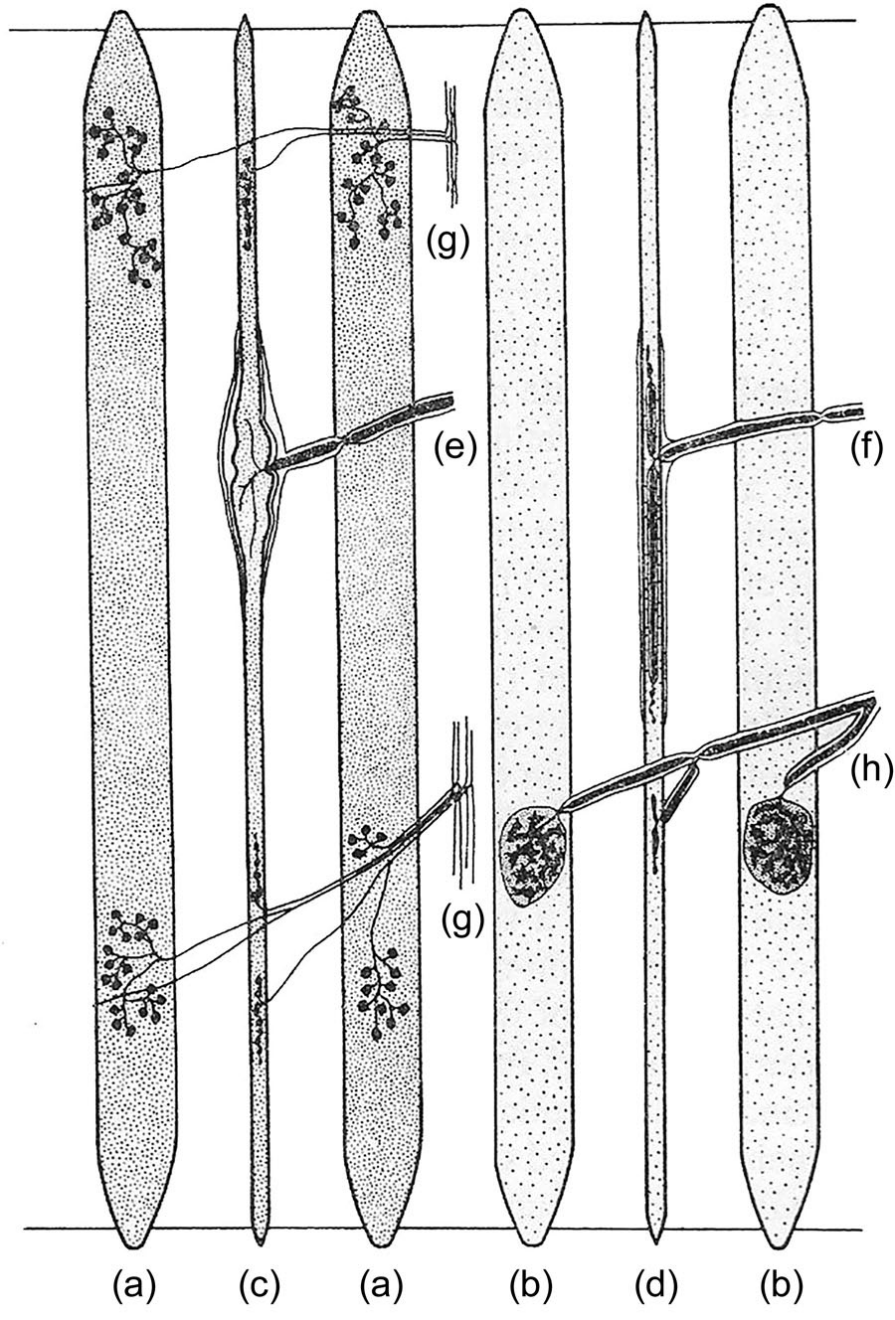
Schematic representations of muscle spindles in lizards. Two spindles are shown, one lying between extrafusal twitch muscle fibres (lightly stippled, b), the other between extrafusal tonic muscle fibres (heavily stippled, a). The spindle comprising the intrafusal fibre (c) has a short‐capsule sensory ending served by sensory axon (e), and it receives motor terminals from collaterals of tonic motor axons (g). The long‐capsule sensory ending supplied by axon (f), lies on intrafusal fibre (d) and receives collaterals of twitch motor axon (h). Redrawn from Proske and Ridge (1974).

Squamate spindles are of two kinds, short‐capsule and long‐capsule, each with a distinctly different sensory terminal. The ending in the long‐capsule spindle may be as long as 1500 µm (Proske, 1969b), whereas in the short‐capsule spindle, it is 80–100 µm (Pallot & Ridge, 1973). In long‐capsule spindles, the afferent axon enters the capsule, where it typically bifurcates, to course for long distances along the surface of the intrafusal fibre (Figure 6). For the short‐capsule ending, the afferent fibre enters the prominent capsule, to divide immediately into several fine branches, each of which comes into close contact with the intrafusal fibre (Figure 6). For both ending types, the sensory region is covered by inner and outer connective and perineurial tissue capsules. In the transverse section at the level of the sensory ending, crescent‐shaped sensory terminals lie in close apposition to the intrafusal fibre.

Ultrastructural details have been provided by Fukami and Hunt (1970) and by Pallot and Ridge (1972, 1973). In the grass snake, Pallot and Ridge (1972) described for long‐capsule spindles a series of bulbous enlargements of the sensory terminal, with narrow links between bulbs, in a similar arrangement to that seen in frog spindles (Katz, 1961). For short‐capsule spindles, the ending branches more profusely, covering much of the area of the intrafusal fibre adjacent to the entry point of the afferent axon into the capsule.

In a squamate spindle, the intrafusal fibre typically has the same length as adjacent extrafusal fibres. Structural studies have shown that there are two kinds of intrafusal fibre; in long‐capsule spindles, the intrafusal sarcomeres have a prominent M line, typical of twitch muscle, whereas in short‐capsule spindles the M line is less distinct, which is typical for tonic muscle. In the region of contact between the sensory ending and the intrafusal fibre of a short‐capsule spindle, there is little myofibrillar material; the myonuclei become more rounded and numerous and the diameter of the intrafusal fibre almost doubles (Fukami & Hunt, 1970). For the long‐capsule spindles, little specialisation of the intrafusal fibre under the sensory ending can be recognised and it has visible striations throughout the sensory region. The patterns of these developments are strongly reminiscent of the specialisations seen in the sensory regions of mammalian nuclear bags and nuclear chain intrafusal fibres.

### 8.2 The motor innervation

**FIGURE 7:**
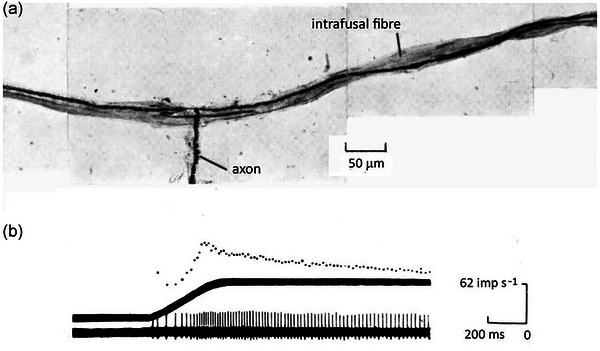
Passive stretch of a long‐capsule spindle in the lizard semimembranosus muscle. (a) Silver‐impregnated, whole‐mount preparation of a long‐capsule spindle showing the sensory axon bifurcating as it approaches the intrafusal fibre, to run long distances in both directions along the intrafusal fibre. (b) Top trace, instantaneous frequency display of afferent response to a 3.5 mm passive stretch of the muscle at 10 mm/s^;^ middle trace, length record; bottom trace, response displayed as action potentials. Redrawn from Proske (1969a, 1969b).

For squamate spindles, as in frog spindles, the motor supply to the intrafusal fibres is from the collaterals of axons supplying extrafusal fibres. Physiological evidence for this in reptiles was provided by Proske (1967). As already mentioned, squamate skeletal muscles consist of twitch and tonic types. Their physiological characterisation has been described by Proske and Vaughan (1968). As for frog muscle, there is some debate about a further subdivision of twitch muscle into slow‐twitch and fast‐twitch types. Certainly, there is evidence that some lizard twitch muscles are multiply innervated (Proske & Vaughan, 1968; Figure 7). Perhaps this is the case for slow‐twitch muscles.

The question of the association between sensory and motor endings for spindles in the two muscle types is controversial. Proske (1969b) reported for a largely twitch muscle (semimembranosus) that it contained only long‐capsule spindles and the intrafusal motor endings were of the twitch type. It remains uncertain whether these were of the slow‐twitch or fast‐twitch types. It was common for the intrafusal fibre to receive more than one twitch motor terminal; often it was two. Such an arrangement may ensure adequate excitation of both poles of the intrafusal fibre in the face of difficulties in transmission of the muscle action potential across the sensory region.

A mixed, slow‐twitch muscle (semitendinosus) had spindles of both the long‐capsule and short‐capsule type. Of 29 spindles counted, 12 were long‐capsule, 17 were short‐capsule. When only one to two motor endings could be located on the intrafusal fibre, and these could be traced back to their parent axon, the extrafusal termination was always on twitch muscle and the spindle sensory ending was of the long‐capsule type. When there were four or more motor endings on an intrafusal fibre, they were supplied by collaterals of extrafusal slow axons and the spindle sensory endings were of the short‐capsule type (Proske, 1969b).

There remains some uncertainty over the association between sensory and motor terminations in squamate spindles. This is because of the difficulty of tracing every intrafusal motor terminal back to its parent axon to identify it as slow or twitch. Nevertheless, looking at the available evidence, the predominant pattern appears to be that the spindle sensory endings follow the pattern of motor innervation; the long‐capsule spindle is associated with twitch muscle and the short‐capsule spindle with tonic muscle. No examples were encountered of spindles with a mixed, slow‐twitch motor innervation (Cliff & Ridge, 1973). A diagrammatic summary of the two types of lizard spindles is shown in Figure 6.

### 8.3 Stretch responses of squamate spindles

Studies were carried out on a predominantly twitch muscle (semimembranosus) and a tonic muscle (scalenus) of the lizard *Tiliqua* (Proske, 1969a). Muscle spindles were identified by their in‐parallel behaviour during muscle contraction. Only occasional tendon organs were encountered in these muscles (Gregory & Proske, 1975). Spindles typically had a resting discharge of 3–20 impulses/s with the muscle shortened to the point where it lay slack. Figure 7 illustrates a long‐capsule spindle from the semimembranosus muscle and its response to a slow stretch. The stretch rate was 10 mm/s.

At this stretch rate, there was a brief, initial burst of impulses at stretch onset, indicating thixotropic behaviour (Proske et al., 1993), and there was only a small peak in rate at the end of the length change, followed by a gradual fall at the new length. Peak firing during the stretch grew larger at faster stretch rates.

**FIGURE 8:**
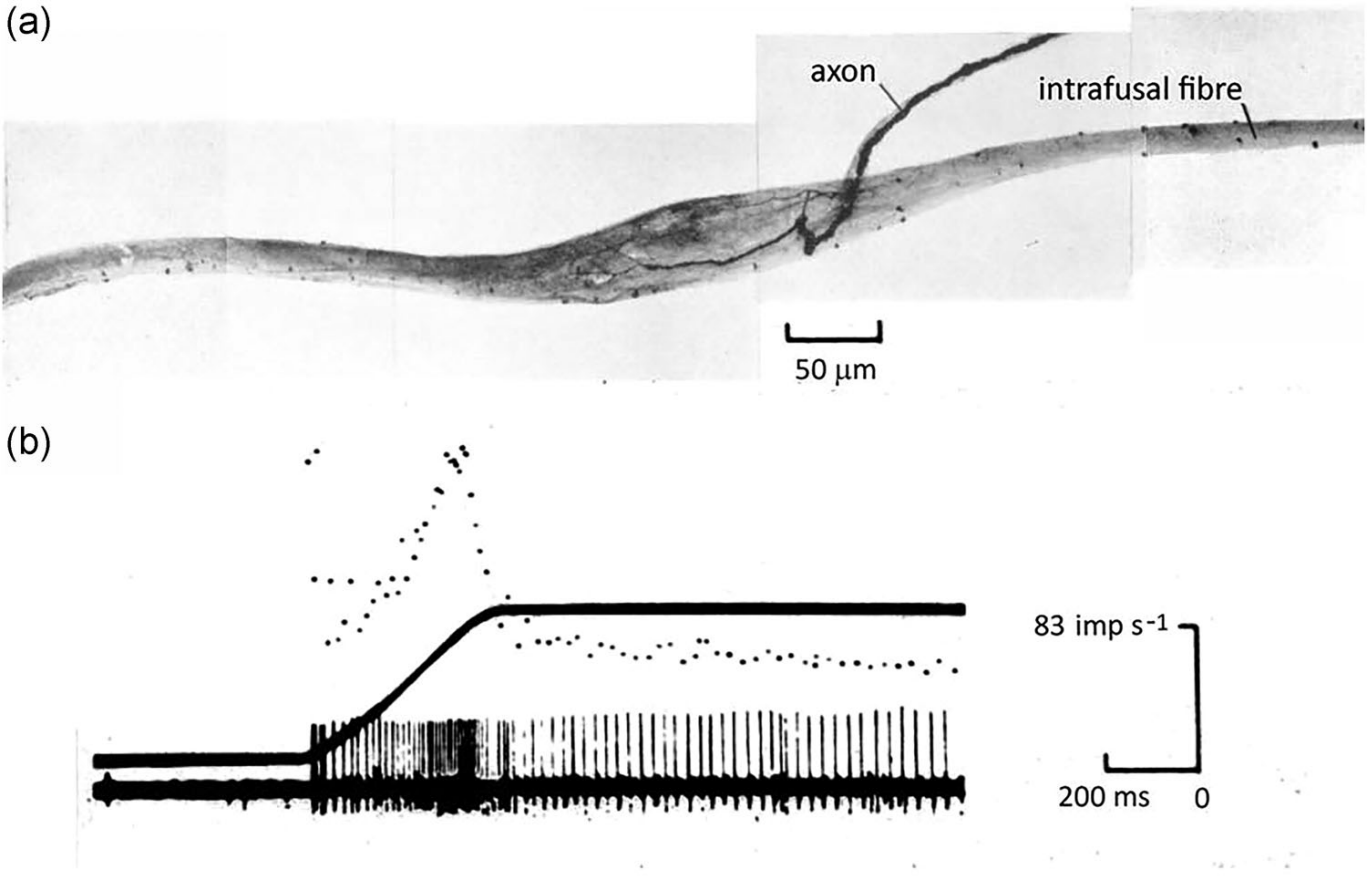
Passive stretch of a short‐capsule spindle in the lizard scalenus muscle. (a) silver‐impregnated whole‐mount preparation of a short‐capsule spindle showing the afferent axon entering the capsule and branching immediately, several times, to terminate within the expanded capsular portion of the intrafusal fibre. (b) Passive stretch response of a short‐capsule spindle to a 5 mm stretch at 11 mm/s. Top trace, instantaneous frequency display of afferent discharge; middle trace, length change; bottom trace, response displayed as action potentials. Redrawn from Proske (1969a,b).

Responses of a short‐capsule spindle from the scalenus muscle are shown in Figure 8. This muscle was believed to contain only short‐capsule spindles. Here, too, there was an initial burst at the onset of the stretch, although this time, it was more prominent, reaching a rate close to the peak response during the stretch. During the length change, the response of the short‐capsule spindle was much larger than for the long‐capsule spindle; the firing rate rose rapidly during the length change, to reach a peak at the end of the stretch. It then fell abruptly to a low level, with a little further adaptation at the new length. To quantify the dynamic responsiveness of the two kinds of spindles, the dynamic index was calculated (peak rate during stretch minus the rate 0.5 s later). This was calculated for each spindle over a range of stretch rates (10–50 mm/s). While there was considerable variation between spindles, the dynamic index increased linearly with the stretch rate for long‐capsule spindles, while for short‐capsule spindles the relation was more than twice as steep for the lowest stretch rates (3–10 mm/s, Proske, 1969a).

Based on the structural specialisations seen in the sensory regions of the two kinds of spindle, it is possible to speculate about how transduction processes might take place. For short‐capsule spindles, the large dynamic response is the result of the sensory ending being in contact with a predominantly elastic element (paucity of myofibrillar material and accumulation of nuclei) that is in series with a more viscous element, the spindle pole. By contrast, the persistence of striations throughout the sensory region and the absence of accumulated nuclei in long‐capsule spindles suggests that here the sensory ending lay on an element which differed little in viscoelastic properties between the sensory and polar regions.

### 8.4 Responses to stretch and motor stimulation

In assessing the influence of motor stimulation on spindle responses, stretch was combined with motor stimulation, similar to the method used with frog muscle (Matthews & Westbury, 1965). This approach derives from the method first used to demonstrate the existence of two kinds of fusimotor fibres supplying mammalian spindles, based on their action during a stretch of the spindle primary ending (Matthews, 1962). Furthermore, impulses from spindles provide the afferent limb of the stretch reflex and are, therefore, important for regulating posture and movement. If the stretch response and its reflex action could be augmented by motor stimulation, this would represent an important contribution by spindles to motor control. Lizard muscle exhibits a stretch reflex (Kenins, 1977).

**FIGURE 9:**
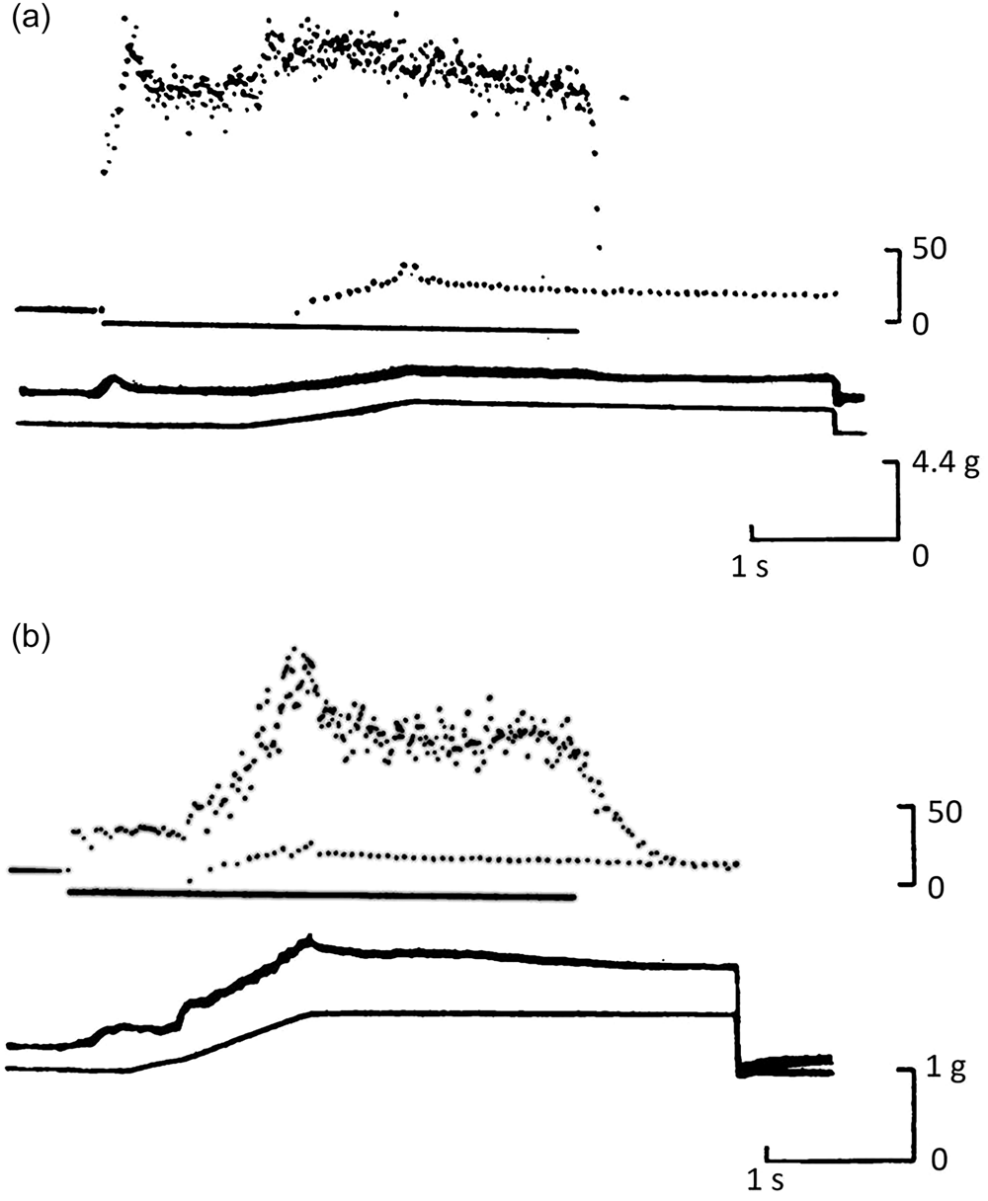
Responses of lizard spindles to combined stretch and motor stimulation. In (a) the lower frequency trace represents the response of a muscle spindle in the fast muscle, semimembranosus, to a passive stretch (1.5 mm at 1.5 mm/s). The top frequency trace shows the response to the same stretch during repetitive stimulation of a twitch motor filament (40 pulses/s). The period of stimulation is shown by the bar below the lower frequency trace. The bottom continuous trace represents the length change, the trace above it muscle tension. The muscle had been partially paralysed with tubocurarine to minimise muscle tension effects on the spindle response during motor stimulation. (b) Spindle from the semitendinosus muscle associated with tonic muscle fibres. Lower frequency trace, response of the spindle to passive stretch (1.4 mm at 1.2 mm/s); upper‐frequency trace, response to combined stretch and stimulation of a tonic motor filament (7 pulses/s). The bar shows the period of stimulation. The bottom continuous trace represents the muscle length change, the trace above it, muscle tension. Again, during stimulation muscle tension had been reduced by adding tubocurarine to the bathing solution. Redrawn from Proske (1973).

The reptilian equivalent of the experiment on frog muscle was carried out on the muscle of the lizard *Tiliqua* (Proske, 1973). Two muscles were studied, semimembranosus, a muscle composed largely of twitch muscle fibres, and semitendinosus, a muscle containing both twitch and tonic fibres. Dissection of filaments of the peripheral nerve isolated functionally single spindle afferents. Other filaments of the nerve were isolated which, on stimulation, had a specific, excitatory effect on the response of the recorded spindle. The influence of stray mechanical effects from extrafusal contractions on the spindle was minimised by adding the neuromuscular blocker tubocurarine to the muscle's bathing solution. Typical examples of responses from the two muscles are illustrated in Figure 9.

Repetitive stimulation of a motor filament (40 pulses/s) acting on a semimembranosus spindle had a powerful excitatory effect (150 impulses/s) on the spindle while the muscle was held at constant length. When the muscle was stretched (1.5 mm at 1.5 mm/s), this produced little further increase in the spindle's response, with no change in rate at the end of the length change (Figure 9a).

The response of a spindle in semitendinosus to stimulation of a tonic motor filament (Figure 9b) showed that the response to motor stimulation (7 pulses/s) at the initial length was rather weak (30 impulses/s). However, during the stretch, spindle discharge increased rapidly to a peak of 150 impulses/s, and at the end of the length change, it fell to a plateau rate of 90 impulses/s, which it maintained for the remainder of the period of motor stimulation. At the end of stimulation, the firing rate fell back slowly to the level the spindle had maintained after a passive stretch. This compares with the much more rapid fall in rate at the end of stimulation in the twitch muscle. Given that each spindle contained only a single intrafusal fibre, these declines in response at the end of stimulation presumably represent the differing relaxation rates of the two intrafusal fibre types.

The two features of spindle responses to tonic‐muscle stimulation were, therefore, the high peak discharge during the stretch and the high, maintained rate at the new length, compared with that at the initial length. The maintained increase was not simply due to a muscle length: tension effect; this was shown by comparing rates in response to motor stimulation during combined stretch and stimulation with stimulation initiated after the stretch had been completed (see Figure 5 of Proske, 1973). The combined response was twice as large as that initiated at the final length.

Similar effects were seen for frog muscle by Matthews and Westbury (1965). However, one feature of responses of frog spindles during combined stretch and tonic motor stimulation was that following the steep fall in spindle discharge rate at the end of the stretch, there was a second, slower decline in rate (Matthews and Westbury (1965); Figure 3d). The authors attributed this decline to the dynamic stimulus rather than representing a progressive fall in the effectiveness of the motor stimulation. A similar slow decline was absent in lizard spindles.

To conclude, combining stretch and motor stimulation produced dramatically different outcomes for the two kinds of lizard spindles; in twitch muscle, intrafusal contraction led to a large response from the spindle, but one that was little altered during a stretch. By comparison, stimulating an intrafusal fibre in a tonic muscle increased the dynamic responsiveness of the spindle.

An explanation for similar motor effects in anuran spindles was provided by Brown (1971b). He suggested that the rate of turnover of cross‐bridges between actin and myosin filaments in sarcomeres of tonic muscle was slower than for twitch muscle, reflecting their respective differences in contraction speed. Stimulating tonic muscle produced relatively low tension while the muscle was at constant length, but during stretch, a larger number of attached cross‐bridges would be stressed at any instant of time, compared with twitch muscle. During stretch, it would, therefore, lead to a proportionately higher tension in the tonic muscle compared with the twitch muscle. Since the intrafusal fibre of a short‐capsule spindle was likely to be of the tonic type, its sensory ending would be sensitised by a lengthening contraction. For the long‐capsule spindle, on the other hand, with the high rate of cross‐bridge turnover in its intrafusal fibre, there would be a large, rapid shortening of the fibre during a contraction, leading to a high static response, but any additional stress on the sensory ending during stretch would be less, due to the high cross‐bridge turnover rate. It is conceivable that a related mechanism is responsible for augmenting the dynamic and static responses of mammalian primary endings during fusimotor stimulation.

### 8.5 Lizard stretch reflexes

Kenins (1977) established that a stretch reflex can be demonstrated in hindlimb muscles of the lizard *Tiliqua*. In a decerebrate preparation, electromyographic and tension recordings during stretch of the muscle, with its nerve supply intact, and after spinal sensory roots were cut, provided an estimate of reflex tension. When expressed as a proportion of active muscle tension, reflex tension for a 1% length change was 3.8–6.0% for *Tiliqua*. This compares with 1.8–6.0% for the soleus muscle of the decerebrate cat (Matthews, 1959).

Therefore, the spinal stretch reflex in *Tiliqua* is of a considerable power, comparable to that in mammals and rather stronger than in frogs (Fadiga & Brookhart, 1960). It was speculated that since in lizards the limbs project laterally from the trunk, rather than downwards, as in mammals, the maintenance of stance would require a strong tonic stretch reflex. Here, the collateral innervation of spindles could provide a means of automatically adjusting the strength of the reflex, particularly if any resistance was encountered during execution of a movement (Kenins, 1977).

Intracellular recordings of spinal motoneurons were achieved in *Tiliqua* and it was shown that in response to muscle stretch, monosynaptic transmission across the muscle afferent–motoneuron synapse could be demonstrated, implying that the stretch reflex could operate at monosynaptic tempo.

## 9 THE ARCHOSAURIA

Of the two living groups of archosaurs, one, the Crocodylia, remain largely unstudied. Brief descriptions of *Alligator* spindles published by Hines (1930) and Cole (1955) show them to consist of a few (2–5; Hines, 1930) intrafusal fibres, enclosed within a capsule apparently with a small periaxial space, containing a single sensory ending, but with no evidence of an exclusively fusimotor innervation. More is known about the spindles of the second group of living archosaurs, the Aves or birds. Here, it might be mentioned that the immediate ancestors of birds were therapsid dinosaurs, bipedal carnivores that were feathered, warm‐blooded and had hollow bones (Pickrell, 2014). The evolutionary step required to achieve powered flight was, therefore, not as great as might have been thought.

## 10 THE AVES

We know relatively little about bird spindles and their physiology. However, one important finding is evidence that bird spindles possess a specific fusimotor supply. This is another example of convergent evolution, given the wide evolutionary separation of birds and mammals (Figure 1). It implies that evolution took a big step in creating what is likely to be a complicated system, involving a degree of selective motor innervation of intrafusal fibres, the establishment of spinal fusimotor neurons and their connections with higher centres in the brain. One possible reason for the emergence of a fusimotor system is the new challenge posed by motor control of bipedal terrestrial locomotion and of powered flight.

### 10.1 Evolution of a fusimotor system

It is, perhaps, worth speculating about possible reasons for the emergence of a fusimotor system in birds. The hypothesis that we propose is based on the fact that birds, like mammals, are advanced endotherms, with both groups exhibiting non‐shivering thermogenesis (Benton, 2021). There is also evidence that dinosaurs and pterosaurs were endothermic, or functionally homeothermic. The carnivorous theropod dinosaurs were likely to have been warm‐blooded (Pickrell, 2014, p. 54). Other, herbivorous, dinosaurs like the long‐necked sauropods, were probably passively homeothermic, as their massive size would have caused them to retain all of the heat they required. We suggest that the energy‐rich diet of the carnivorous theropods led to the development of endothermy. This was taken advantage of by birds, which used it to expand their home ranges into areas previously inaccessible to them. At the same time, the maintenance of a high and constant body temperature allowed birds to become behaviourally much more active than other diapsids. Here, a consideration is powered (rather than gliding) flight. To be able to fly did mean tight constraints on body size, but that, in turn, made it easier to execute fine movements. We propose that for the execution of sophisticated movements, as seen in birds, peripheral sensory support from muscle spindles controlled by a collateral motor innervation was no longer adequate. In other words, the opportunity provided by endothermy for the execution of more complex movements was exploited by the development of a fusimotor system. Presumably, something similar had occurred in early mammals, which ultimately reached its peak in the fine movement control observed, for example, in primates.

### 10.2 Structure and function of bird spindles

**FIGURE 10:**
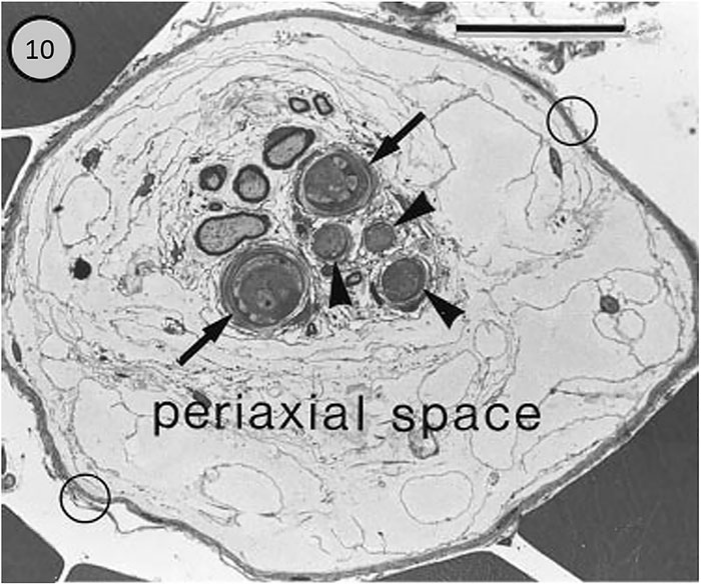
Transverse section through the sensory (equatorial) region of a spindle from posterior latissimus dorsi of the domestic (Leghorn) chicken. The multilayered outer capsule (open circles) encloses a large periaxial space, surrounding two large (arrows) and three small (arrowheads) intrafusal fibres, some with prominent sensory terminals, and several profiles of the myelinated branches of the sensory axon. An inner capsule of thin, flattened, fibroblast‐like cells is present, enclosing individual intrafusal fibres. (Scale bar = 20 µm, light micrograph, toluidine blue, from Ovalle et al. (1999).

The avian spindle has been reviewed by Barker (1974) and Maier (1992). The account by Maier lists descriptions of spindles in 13 species, ranging in size from the zebra finch to the turkey, and 24 named muscles mostly from the wing and leg. Despite this range of source data, there are many common features (Figure 10). The outer capsule is well developed; like all other vertebrate muscle spindles, it is continuous with and structurally similar to the multilayered perineurium of the supplying nerve. It is expanded in the sensory (equatorial) region of the intrafusal fibres, enclosing a prominent periaxial space. In the polar regions, it closely invests the intrafusal muscle fibres, which usually extend well beyond the limits of the capsule. A much shorter inner capsule, apparently composed of fibroblast‐like cells as in mammals, surrounds each intrafusal fibre and encloses the sensory terminals (Hikida, 1985).

Perhaps the most variable feature of avian spindles is the number of intrafusal muscle fibres. The extremes of the ranges for the complete sample listed by Maier (1992) are 1–22. Spindles with only one or two intrafusal fibres are more common in the smaller species (zebra finch and canary), but they are not uncommon in larger species such as chicken and duck (Adal & Chew Cheng, 1987; Maier & Eldred, 1971). There have been conflicting reports on the morphological classification of the intrafusal fibres into sub‐types, probably reflecting the different species and muscles studied, but there is now clear evidence from electron microscopy, histochemistry and morphology for at least two, and possibly three, types of fibre – a fast twitch type and one or two slow types, though in complete contrast to the mammalian spindle, the fast type tends to be larger in diameter than the slow type(s). Equatorial nuclei do occur in avian intrafusal fibres, though there are far fewer than in mammalian spindles, and they typically present a myotube‐ or nuclear‐chain‐like appearance in all fibres. Quantitative differences in the distribution of satellite (stem) cells and myocytes described in the anterior and posterior latissimus dorsi muscles of the chicken (Kirkpatrick et al., 2008) are probably related to the smaller eventual size of intrafusal, as compared to extrafusal, fibres.

The most informative study of the innervation of avian spindles is that of Chin (1970), who combined cholinesterase and silver impregnation techniques to examine teased, whole spindles from anterior and posterior latissimus dorsi muscles of the chicken. Almost invariably there was a single large‐diameter afferent axon supplying all of the sensory terminals in each equatorial region. If this is true of avian spindles in general, then secondary endings are probably absent from all amniotes other than mammals. A similar conclusion was reached by Adal and Chew Cheng (1980). Although the form of the sensory terminals has been described as annulospiral (Ovalle et al., 1999), it is more usually described as comprising ‘bulbs or varicosities linked by rather fine neural connections’ (Adal & Chew Cheng, 1980).

For duck spindles, Chin (1970) identified small‐diameter axons entering the spindles as motors. Often, a single motor axon accompanied the afferent before branching extensively to form simple, multiterminal or ‘grappe’ endings in regions on either side of the sensory ending and close to it. In some cases, at least, the axon appeared to supply endings to all of the intrafusal fibres. Individual spindles might receive several such axons with distributions extending into polar regions. Other, small‐diameter, motor axons tended to be more sparsely branched with ‘plaque’ or plate‐like endings usually in more polar locations. Chin (1970) reported that no axon supplied both grappe and plaque endings, and that only grappe endings occurred in spindles of the anterior latissimus dorsi muscle, which was exclusively composed of slow extrafusal muscle fibres, whereas both types of ending could be found in spindles of the fast twitch posterior latissimus dorsi. Curiously, in spindles with only one intrafusal fibre, both types of ending could be present. In addition, a few examples of intrafusal grappe or plate endings were seen to derive from branches of axons supplying extrafusal muscle fibres, representing a collateral innervation.

**FIGURE 11:**
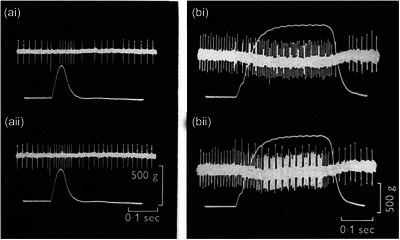
Responses of a spindle from the lateral gastrocnemius muscle of the domestic duck to (a) single shock and (b) repetitive stimulation of the muscle nerve at 45 pulses/s. In each panel, the upper trace is spindle impulse activity, the lower trace, muscle tension. (ai) Stimulus strength 1.4 times threshold, which evoked a maximal twitch. (aii) Stimulus strength increased to 2.1 times threshold to recruit fusimotor‐evoked impulses on the rising phase of the contraction. (bi) Maximal tetanic stimulation; (bii), stimulus strength increased to 10 times maximum to recruit additional impulses. The larger spikes during stimulation in (bii) are stimulus artefact‐direct spike complexes. Re‐drawn from Dorward (1970).

Perhaps the most important finding for birds is that spindles possess a specific fusimotor innervation, as shown by Dorward (1970), who used the desheathed sciatic nerve of the domestic duck split into fine filaments for recording and stimulation. Muscle spindle afferents were identified by their unloading response during muscle contraction (Figure 11ai). Afferent conduction velocity distribution was unimodal, consistent with Chin's (1970) conclusions.

Dorward's measurements, made at 40°C, gave an afferent conduction velocity range of 24–66 m/s, which lies within the mammalian (feline) group II range. Unfortunately, we do not know what kinds of stretch responses these afferents generated. The presence of a specific fusimotor innervation of spindles in several muscles was demonstrated by stimulating selected motor filaments. In Figure 11ai can be seen the unloading response of the spindle discharge by a maximal muscle twitch. When stimulus strength was increased further (Figure 11aii), additional impulses were recruited during the contraction without any accompanying change in recorded tension. During repetitive stimulation, the afferent response during a maximum contraction (Figure 11bi) was increased significantly by increasing stimulus strength 10‐fold (Figure 11bii). These effects were attributed to the recruitment of fusimotor fibres.

In addition, Dorward (1970) presented evidence that stimulation of larger efferent axons, at higher rates than required to elicit maximal tetanic tension, could sometimes produce increased afferent responses, indicating the presence of a collateral motor innervation of spindles by these skeletomotor axons.

## 11 THE SYNAPSIDA

The two groups comprising the Amniota are the Diapsida, discussed in the previous sections, and the Synapsida. The synapsids can be broken down into three further groups, the Monotremata, the Metatheria or marsupials, and the Eutheria or placental mammals. Relatively little is known of the structure and innervation of monotreme and marsupial spindles, but what has been described is cited, in the hope that future studies will provide a more comprehensive picture.

### 11.1 The monotremes

There are three living groups of monotremes, the platypus (*Ornithorhynchus*), the echidna (*Tachyglossus*), both found in Australia, and *Zaglossus*, the long‐nosed echidna of New Guinea. The one account of monotreme spindles comes from Voss (1963), who posed the question of whether monotreme spindles were reptile‐like with only a single intrafusal fibre or were more representative of mammals in having multiple intrafusal fibres (at the time spindles in the Testudines, with their multiple intrafusal fibres, had not yet been described). In material from the neck and shoulder muscles of the echidna, Voss described the presence of spindles with thin capsules and no apparent periaxial space. The spindles were 1.5–2.5 mm long, which was only half the length of the adjacent extrafusal fibres. Each spindle contained six to eight intrafusal fibres.

In platypus, a similar number of intrafusal fibres was counted for each spindle. However, the spindles were rather shorter than in the echidna, at 0.6–1.2 mm long. On the basis of this rather limited evidence, Voss concluded that for the two groups of monotreme spindles examined, their structure suggested that they were related more to placental mammals than to reptiles.

### 11.2 The marsupials

The main account of the structure of marsupial spindles comes from Jones (1966a, 1966b). For material from a variety of leg and toe muscles of the Australian brush‐tailed possum, *Trichosurus*, he described each spindle as containing one to three nuclear‐bag intrafusal fibres and 2–16 nuclear‐chain intrafusal fibres, a dual classification widely accepted for placental mammals at that time (Matthews, 1964). The identification of the two fibre types was based on their size and length, the bag fibres being larger and longer than the chain fibres, and showing an accumulation of nuclei in the capsular region. The chain fibres had nuclei arranged in a longitudinal chain.

There were two types of sensory endings. The primary ending consisted of a series of tight spirals around both nuclear‐bag and nuclear‐chain intrafusal fibres, over a length of 150–250 µm. These endings occupied the equatorial region of the intrafusal fibres. The mean diameter of afferent fibres supplying the primary ending was 8–15 µm with a peak of 11–13 µm. This fell within the diameter range for afferents supplying the primary endings of placental spindles.

About one‐half of spindles had secondary endings. Axons supplying secondary endings had an average diameter of 8 µm. The average length of a secondary ending was 200–300 µm. The afferent fibre is typically divided when approaching the spindle, and each branch clasps the intrafusal chain fibres in a juxta‐equatorial position with a series of incomplete spirals. There were also terminals on a small portion of the myotube region of nuclear‐bag fibres.

All spindles received motor axons with a diameter of 1–2 µm. These were referred to as ‘small’ motor axons. Nuclear‐bag and nuclear‐chain intrafusal fibres received a separate ‘small motor fibre’ innervation. In addition, one‐third of identified spindles received one or more motor axons in the 2.5–4.5 µm range. These were referred to as ‘large’ motor axons. The distribution of axon diameters for identified α‐motoneurons was given as 3.0–6.5 µm. In the most parsimonious interpretation of the data, the ‘small’ motor axons were γ‐motoneurons and those spindles additionally supplied by ‘large’ axons were receiving a collateral motor innervation by branches of α‐motoneurons. Since it was not possible to trace the origin of the ‘large’ motor axons beyond 1 mm from the spindle, this could not be established with certainty. However, Berndt et al. (1969) reported a collateral motor innervation in spindles of the American opossum (marsupial). In comparison with reptilian muscle (*Boa constrictor*), the opossum showed an incidence of two examples of a collateral innervation in 159 spindles studied, while in the reptile the incidence was six examples in 40 spindles. That the ‘small’ motor axons, referred to by Jones (1966a), are γ‐motoneurons seems likely but needs confirmation by physiological studies.

### 11.3 The Eutheria

Most of our knowledge of the organisation and function of muscle spindles, including their role in motor control, has been obtained from study of the eutherian, or placental, mammals especially the terrestrial and cursorial species. Indeed, a whole monograph has been dedicated to the subject (Matthews, 1972) and, more recently, several reviews have been devoted to particular aspects of spindle biology (Banks, 2005; Banks & Barker, 2004; Proske & Gandevia, 2012; 2018). As mentioned above, spindles are now believed to subserve a wide range of functions, ranging from a role in proprioception to wound healing. Knowledge of many of these roles is limited to placental mammals, simply because the relevant studies have not yet been carried out on other animals, or are unable to be performed on them. They will, therefore, not be considered in detail.

### 11.4 Structural considerations

**FIGURE 12:**
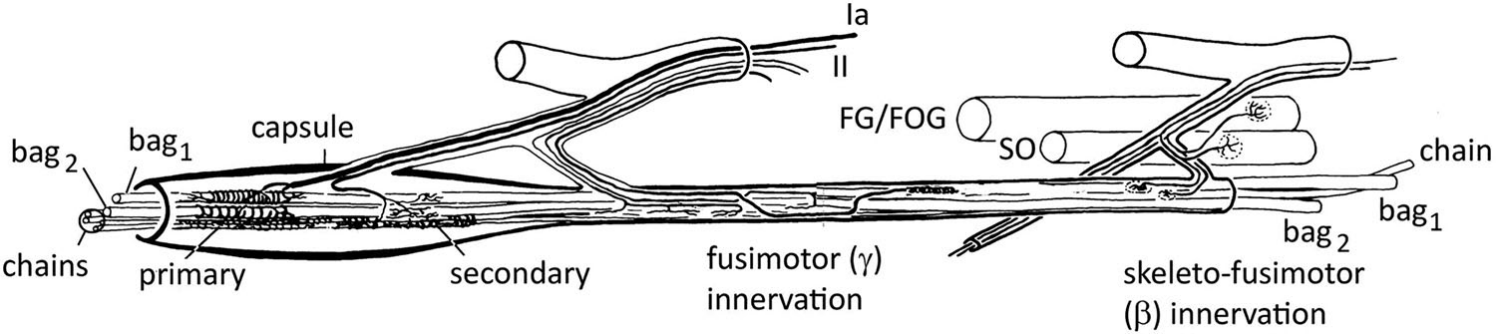
Semi‐schematic diagram to illustrate the structure and innervation of a eutherian muscle spindle. Only the sensory (equatorial) region and the encapsulated portion of one polar region are shown. Typically, the single primary sensory ending has terminals on all three kinds of intrafusal muscle fibre, bag_1_, bag_2_ and chains. It is supplied by a group Ia afferent, one of the largest diameter axons of the body. In addition, one or more secondary sensory endings, supplied by smaller group II afferents, may be present within the expanded portion of the capsule. Axons providing a specific fusimotor (γ) innervation usually enter the spindle together with the sensory innervation. A skeleto‐fusimotor innervation, sometimes known as β‐innervation, is present in some spindles, the intrafusal endings being supplied by collaterals of motoneurons innervating extrafusal muscle (skeletomotor, or α‐innervation). Modified from Banks and Barker (2004).

Spindles of placental mammals exhibit a basic unity of structure (Figure 12), with variations that are related to body size and to the particular muscles and muscle groups that contain them (Andrew et al., 1973; Banks, 2005, 2006; Banks et al., 2009). The capsule, as in all vertebrate spindles, is multilayered, continuous with, and evidently derived from perineurium of the nerve supply. In placental mammals, the capsule is particularly extensive, enclosing most, if not all, of the spindle's nerve supply. The extent of the fusiform enlargement and its prominent periaxial space depends on the number of sensory endings it contains. As in birds, there is an inner capsule of fibroblast‐like cells that wrap around the sensory terminals on each intrafusal fibre without contacting them directly.

There are three types of intrafusal fibre, initially characterised by a combination of ultrastructural and histochemical properties (Banks et al., 1977), and more recently by the application of immunohistochemistry, especially to localise different myosin heavy chain (MyHC) isoforms (Thornell et al., 2015). In general, the bag fibres are longer and have greater diameters than chain fibres, though again, there is variability between muscles and species. In the smaller animals (mouse, rat, guinea pig, rabbit) the most common complement of intrafusal fibres is one bag_1_, one bag_2_, and two or three chain fibres. In larger species, including humans, there may be up to eight or nine chain fibres. It is known that bag_1_ fibres have properties reminiscent of extrafusal slow (tonic) muscle while chains are rapidly contracting twitch fibres and bag_2_ fibres are intermediate in their properties. So, for example, both bag_1_ and bag_2_ fibres, but not chain fibres, are able to undergo tonic contractures in response to the depolarising drug suxamethonium, a feature of slow muscle (Taylor et al., 1994).

In each of the three types of intrafusal fibre the equatorial, or sensory, region is characterised by the replacement of most of the contractile structure present throughout the rest of the fibre by a group of euchromatic nuclei, leaving only a thin outer rim of myofibrillar material. The presence and arrangement of these nuclei have been known for decades, yet the functional significance of so many nuclei in such a small cellular space has rarely been addressed. Banks (2024) suggested that they may play a mechanical role as three‐dimensional springs. While speculative, it is consistent with the behaviour of the equatorial region, whose tension under steady‐state conditions in the passive spindle increases linearly with length, whereas that of the polar regions increases exponentially with length. The equatorial region, therefore, behaves like a pure spring with constant stiffness, whereas the polar regions progressively increase in stiffness as they are lengthened (Poppele et al., 1979).

Our most complete information on spindle innervation, once again, has been obtained from studies on the cat, but it is important to emphasise that the very high degree of differentiation seen in cat spindles is not found in all placental mammals. As in their sister group, the marsupials, the spindles of placental mammals may each contain more than one sensory ending, the average number of endings being characteristic for different muscles, and most probably for different species. The actual number in any single spindle is, however, determined randomly during development (Banks & Stacey, 1988). The great majority of sensory endings of cat spindles are readily distinguishable as either primary or secondary. The spindle's equatorial region is normally occupied by the terminals of a single afferent axon from the largest group (Ia). The terminals are distributed to all the intrafusal fibres where they adopt open‐loop and spiral forms. Their longitudinal extent is closely correlated with the location of the equatorial nuclei.

As we have seen above, spindles in squamate reptiles are of two kinds that differ in the responsiveness of their sensory endings; but in marsupial and placental mammals there may be two distinct sensory terminals, the primary ending and the secondary ending, enclosed within the same spindle capsule. The primary ending is responsive both to the muscle length change and the rate of length change. The secondary ending, on the other hand, while also responsive to the length change, has little rate sensitivity. Although the primary ending and its Ia axon are essential for the development of the muscle spindle, and consequently are always present, there may be present an additional 0–6 secondary endings (Banks et al., 2009). Secondary endings are located on either side of the equatorial region and clearly have some morphogenetic influence on intrafusal‐fibre diameter. This is especially so for the chain fibres, which receive most of the secondary‐ending terminals and which remain smaller in diameter in the sensory region, compared with the adjacent polar region. They also possess some central nuclei. For further details on secondary endings, see the recent review by Banks et al. (2021).

### 11.5 Skeleto‐fusimotor (‘β’) innervation

For a review of the motor innervation of mammalian spindles, see Banks (1994). We have seen above that the motor supply to amphibian and reptilian spindles has been described as a collateral innervation; a motor axon may divide to innervate both intrafusal and extrafusal muscle fibres. This means that whenever the muscle contracts, its spindles are excited as well, and any spindle‐evoked reflex action is able to reinforce the contraction. As far as we know, in these animals the spindle motor innervation is provided exclusively by collaterals of skeletomotor neurons. When the spindle lies in the tonic muscle, the intrafusal contraction tends to be slow, and this enhances the dynamic sensitivity of spindles; when it is in twitch muscle, it tends to be fast and exerts a static action. Such a collateral innervation also occurs in mammals where it is often referred to as ‘β’ or skeleto‐fusimotor (Bessou et al., 1965). This so‐called β‐innervation can be static or dynamic in its action on spindle stretch responses. For the mammalian β‐system, when muscle composition includes a majority of type S (slow twitch) motor units (Burke et al., 1973), the β action tends to be dynamic; when the majority of motor units is of type FR (fast, fatigue resistant), more incidences of β static effects are encountered (Emonet‐Dénand et al., 1992).

The central motor command for initiating a contraction is normally accompanied by fusimotor activity sufficient to ensure spindle discharges increase rather than decrease during the contraction (Vallbo, 1971). If skeletomotor and fusimotor neurons are commonly co‐activated, it raises the question, why improve on a collateral innervation of spindles, as seen in amphibians and reptiles? The objective may be to maintain spindle firing at much the same rate during a movement, when it is proceeding according to plan, and that is best done by modulating the ongoing static fusimotor activity. Under those conditions, spindles are able to ensure prompt compensatory action in the face of unexpected deviations of the movement from its course. It suggests that to ensure a desired outcome for the planned movement the independence of the fusimotor system from the skeletomotor pathway would provide a degree of flexibility to allow adjustments to be made that would not be available with a collateral innervation.

### 11.6 Fusimotor (‘γ’) fibres

Dynamic axons, whether γ or β, exclusively innervate the bag_1_ fibre and the heightened dynamic sensitivity of the spindle during dynamic fusimotor stimulation is attributable to activity coming from the bag_1_ fibre. Static γ‐axons excite bag_2_ and chain fibres, either separately or together, and some chain fibres are innervated by static β‐axons. The static γ or β activity influences the muscle length sensitivity of spindles. Dynamic fusimotor stimulation influences only the primary ending of the spindle while static fusimotor stimulation can influence both primary and secondary endings. It is thought that secondary endings provide a misalignment signal between the actual and the commanded muscle length while primary endings respond as much to the velocity of the length change as upon the absolute value of muscle length. Thus, an increase in dynamic fusimotor stimulation is unable to prevent the unloading effects of any accompanying extrafusal contraction, while static fusimotor stimulation is able to do so.

A comprehensive discussion of the reflex and other central actions of spindles is beyond the scope of the present review, but one fundamental observation made 70 years ago by Hunt (1952) illustrates the importance of a separate, exclusively fusimotor control of spindle excitability in regulating proprioceptive feedback. In the decerebrate cat, Hunt cut the ventral‐root motor supply to a muscle (gastrocnemius) and in the peripheral, cut end of the root, he isolated 28 single, functional fusimotor fibres supplying gastrocnemius spindles. At the same time, he recorded motoneuron responses from the central, cut end. Repetitive stimulation of the collection of fusimotor fibres exerted a powerful excitatory effect on spindles in the muscle to the point that it initiated reflex responses in motoneurons, probably including at monosynaptic latency. Interestingly, combined stimulation of the fusimotor axons did not generate any measurable muscle tension. Presumably, the force generated by the contracting pool of intrafusal fibres was directed exclusively at exciting the spindles, and the intrafusal attachments to adjacent extrafusal fibres were too compliant to generate measurable muscle tension.

A clue about the role of the fusimotor system in locomotion comes from spindle recordings made in freely moving animals (Prochazka et al., 1976). During voluntary stepping movements, the recorded pattern of spindle activity from limb muscles suggested a low level of γ static activity which closely followed the muscle length changes, signalling that the movement was proceeding according to plan. However, if the animal's limb was unexpectedly grasped and stretched, a heightened dynamic sensitivity was revealed, suggestive of γ dynamic activity. These observations suggested that the fusimotor system was able to switch rapidly from a static to a dynamic fusimotor bias, depending on the changing circumstances (Prochazka et al., 1985).

### 11.7 Responses of passive spindles

Muscle spindles are stretch receptors. They respond to muscle stretch with a length‐dependent increase in maintained rate of impulse discharge and this is believed to be interpreted by the brain in terms of a length change in the receptor‐bearing muscle, that is, a change in the angle at the joint at which the muscle is acting. Muscle spindles are unique among sensory receptors in being contractile and, therefore, able to modulate their stretch sensitivity. While many aspects of the responses of spindles have been revealed, a comprehensive picture of the role of spindles in motor control remains elusive.

While mammalian spindles are independently contractile in that they have a fusimotor supply to the intrafusal fibres, passive spindles also have important functional roles. Their resting activity contributes to the senses of position and movement of the limbs and trunk (proprioception). Raising levels of resting discharge in spindles produces local, segmental reflex inhibition (Hultborn et al., 1996; Wood et al., 1996). Furthermore, it is known that some cells in the primary somatosensory area (S1) of the cerebral cortex are selectively sensitive to passive rather than active movements (London & Miller, 2013).

The bag_2_ fibre is typically the largest intrafusal fibre within the spindle and there is some evidence that, in the absence of any fusimotor activity, the sensory terminals on this fibre are responsible for the spindle's resting discharge (Proske et al., 1991). Occasionally, a spindle can be encountered without a dynamic fusimotor supply. The passive stretch response of such spindles, which presumably lack a bag_1_ intrafusal fibre, is indistinguishable from that of a spindle with its dynamic fusimotor supply present (Gioux et al., 1991). Therefore, in the passive spindle, the evidence supports the view that both the resting activity and the stretch response arise in the bag_2_ fibre, that is, the passive spindle behaves as a single intrafusal fibre spindle (Proske, 1997).

### 11.8 The stretch reflex

In the present account we have repeatedly mentioned the stretch reflex because it is a fundamental reflex response for which the spindle is believed to be responsible and about which a little is known in non‐mammalian vertebrates. However, this is not to imply that it is the only, or even the principal, role of the spindle. Most other aspects of spindle function remain untested in non‐mammalian vertebrates.

The tonic stretch reflex is believed to represent the neural basis of skeletal muscle tone, the resistance to passive movement of a limb in the conscious animal. The tendon jerk is a phasic manifestation of the stretch reflex. In the decerebrate cat, the reflex response to stretch includes both tonic and phasic components. When the application of the stretch is confined to only a portion of the muscle, the reflex contraction is restricted to that portion. The stretch reflex is a resistance reflex; it resists disturbances from a given setpoint and sensitivity of the reflex is several times greater in response to small disturbances compared with larger ones. It means that the reflex is able to resist large limb displacements without being driven into saturation. Reflex tension can be generated both by stretch and by muscle vibration, a stimulus that powerfully excites the primary endings of spindles. Typically, the stretch response is larger and in response to combined stretch and vibration, there is no evidence of occlusion. It suggests that during stretch, there is a contribution to reflex tension from both spindle primary and secondary endings, given that secondary endings are relatively insensitive to vibration (Matthews, 1972).

The increase in responsiveness of spindle primary endings during dynamic fusimotor activity may help to maintain the sensitivity of the stretch reflex in the face of external disturbances. The most important parameter is sensitivity to small disturbances. This allows the maintenance of reflex tone during a static holding action. Dynamic fusimotor activity will also increase sensitivity to large stretches, which would help the muscle resist such stretches and, therefore, maintain reflex stability.

The action of static fusimotor fibres tends to lower the velocity sensitivity of the spindle primary ending and, therefore, its sensitivity to small perturbations. A large, rapid movement may saturate the afferent impulse mechanism of the spindle primary ending and a reduction in sensitivity may play a role in the static fusimotor system in helping to avoid this. The activity of static fusimotor fibres is able to maintain activity in both primary and secondary endings during large muscle shortenings. Preventing silencing of the primary ending would allow servo‐assistance during large, rapid movements (Matthews, 1972).

### 11.9 Other spindle functions

As already mentioned, knowledge of many aspects of spindle function is restricted to mammals, and therefore, a comparison with sub‐mammalian vertebrates is not possible. However, it is worth mentioning some of these roles, to emphasise the breadth of the subject. Presumably, as animals evolved, and their movements became ever more complex, it was necessary to expand the range of functions spindles were required to subserve.

An important clue about spindle function comes from the central projection pathway taken by spindle afferents. The main projection for the hind‐limb is the dorsal spinocerebellar pathway. For the forelimb, it is slightly different, but it conforms to the same pattern. The second‐order neurons terminate in the anterior cerebellum with collaterals projecting to the brainstem. The brainstem pathway continues to thalamus and cortex. The cerebellar and cortical terminations have led to the suggestion of two kinds of proprioception, conscious and unconscious. The cortical pathway is thought to be concerned with conscious proprioception; our ability to determine the position and movement of our body parts, which ultimately give us our sense of self‐awareness. The cerebellar destination, concerned with unconscious proprioception, is thought to involve a role in motor planning and execution; arm position is sensed based on incoming afferent signals plus predictions derived from previous motor commands and a forward model. In addition, roles have been proposed for spindles in the sense of effort (Luu et al., 2011) and their responsiveness is subject to cognitive and emotional factors acting through the fusimotor system (Ribot‐Ciscar and Ackerley, 2021).

## 12 CONCLUDING COMMENTS

Based on phylogenetic evidence, peripheral sense organs structurally recognisable as spindles probably first appeared in early Amniotes as they became fully terrestrial. Then, perhaps much later, similar sense organs originated separately in the semi‐aquatic Anura. This, therefore, is one example of convergent evolution. It is likely that in the Anura spindles were associated with the animals moving out of the water to begin a semi‐terrestrial existence. It may be that spindles in early Amniotes underwent a similar process. One ancestral sensory ending that may well have been a precursor to anuran spindles was the extrafusal receptor seen in both anurans and urodeles.

The second case of convergent evolution encountered in this review was the separate appearance of a fusimotor innervation in birds and mammals, underlining its importance for motor control. The presence of a fusimotor innervation in birds and mammals presumably reflects the growing sophistication of limb movement skills in mammals and of bipedalism and powered flight in birds, these, perhaps, having been facilitated by the prior evolution of endothermy (Benton, 2021). These two examples of convergence, the appearance of spindles in two disparate groups and the emergence of a fusimotor innervation in birds and mammals, provide a hint of the selection pressures that must have operated to allow the evolution of such a major change and, therefore, for the significant advantages this brought to the animals who acquired them.

Perhaps the defining feature of spindles was the development of a cellular and connective tissue capsule, which helped to protect sensory endings on the intrafusal fibres from mechanical disturbances exerted by adjacent extrafusal contractions. Looking across spindles in different vertebrate groups, the number of intrafusal fibres per spindle appears to be quite variable, ranging from 1 to 22. Presumably, the upper limit in the number is determined by the number and branching capacities of the afferent fibres, as they divide, to supply terminals on each intrafusal fibre. The mechanical properties of the intrafusal fibres of ancestral spindles appear to have been determined by their extrafusal environment, whether they lay in twitch or tonic muscle. That, in itself, suggests that the original function of spindles was to provide a supporting response to extrafusal muscle contractions, perhaps by acting through the stretch reflex. A similar trend is followed with the emergence of two kinds of sensory endings, seen in squamate reptiles. Here, the intrafusal fibre contractions are matched to the sensitivities of the sensory endings, to enhance further the spindle's supporting role for reflex contractions. The emergence of a specific fusimotor supply in birds and mammals brought with it the advantage of selective motor control of the spindle, independent of the control of extrafusal muscle. Evolution of a fusimotor supply has arisen independently in birds and mammals. At least in mammals, there are static and dynamic fusimotor fibres acting on three kinds of intrafusal fibres and their sensory endings, primary and secondary. Sensory and motor supplies to the spindle are brought together to lie within one capsule. More is known about muscle spindles in placental mammals than for any other animal group and the range of functions of this receptor has been revealed to be astonishingly diverse. It suggests that as animals evolved new motor skills, this was accompanied by a diversification of the roles of spindles.

## 13 FUTURE DIRECTIONS

Looking to the future, the present review has raised a number of issues. It would be interesting to know more about the transition of early quadrupeds from living in shallow water to living on land. As yet, the fossil record provides little evidence about the existence of terrestrial locomotion amongst the early quadrupeds (Clack, 1997). This is an important gap in our knowledge of the evolution of locomotion.

Another important area for future research concerns muscle spindles in birds. We know the size distribution of their afferent fibres is unimodal. This suggests that afferents are not separable into primary and secondary endings, as in mammals. However, it does not exclude the possibility of two types of responses to static and dynamic changes in muscle length by afferents of similar calibre, as is the case in squamate reptiles. We also lack important details about the avian fusimotor system. Dorward's (1970) observations suggest, based on stimulus threshold differences between skeletomotor and fusimotor axons, that fusimotor fibres of birds, like those in mammals, are significantly smaller than skeletomotor axons. This needs confirmation. An important question for the future is whether avian fusimotor fibres are separated into static and dynamic types. A specific fusimotor system may have first appeared in birds, but just how closely does it resemble the mammalian fusimotor?

We have linked the emergence of a fusimotor system with the appearance of endothermy in animals. It will be important to further strengthen this hypothesis. It would be helpful to have a correlation between ambient body temperature and movement speed for animals thought to be transitional forms in the establishment of endothermy. Perhaps Benton (2021) is right in stating that after the Permian–Triassic mass extinction, surviving synapsids and archosaurs that had previously been sprawlers both began to adopt an erect gait. The new posture, coupled with endothermy, allowed them to move faster through their environment and so compete more effectively for food resources.

## AUTHOR CONTRIBUTIONS

Both authors have read and approved the final version of this manuscript and agree to be accountable for all aspects of the work in ensuring that questions related to the accuracy or integrity of any part of the work are appropriately investigated and resolved. All persons designated as authors qualify for authorship, and all those who qualify for authorship are listed.

## CONFLICT OF INTEREST

None declared.

## FUNDING INFORMATION

None.
